# The Structural Basis of the Binding of Various Aminopolycarboxylates by the Periplasmic EDTA-Binding Protein EppA from *Chelativorans* sp. BNC1

**DOI:** 10.3390/ijms21113940

**Published:** 2020-05-30

**Authors:** Kevin M. Lewis, Chelsie L. Greene, Steven A. Sattler, Buhyun Youn, Luying Xun, ChulHee Kang

**Affiliations:** 1 Department of Chemistry, Washington State University, Pullman, WA 99163, USA; kevin_lewis@wsu.edu (K.M.L.); chelsie.boro@wsu.edu (C.L.G.); 2 School of Molecular Biosciences, Washington State University, Pullman, WA 99163, USA; s.andrew.sattler@gmail.com; 3 Department of Biological Sciences, College of Natural Sciences, Pusan National University, Busan 46241, Korea; bhyoun72@pusan.ac.kr

**Keywords:** bioremediation, EDTA, periplasmic binding protein, crystal structure, isothermal titration calorimetry, ABC transport

## Abstract

The widespread use of synthetic aminopolycarboxylates, such as ethylenediaminetetraacetate (EDTA), as chelating agents has led to their contamination in the environment as stable metal–chelate complexes. Microorganisms can transport free EDTA, but not metal–EDTA complexes, into cells for metabolism. An ABC-type transporter for free EDTA uptake in *Chelativorans* sp. BNC1 was investigated to understand the mechanism of the ligand selectivity. We solved the X-ray crystal structure of the periplasmic EDTA-binding protein (EppA) and analyzed its structure–function relations through isothermal titration calorimetry, site-directed mutagenesis, molecular docking, and quantum chemical analysis. EppA had high affinities for EDTA and other aminopolycarboxylates, which agrees with structural analysis, showing that its binding pocket could accommodate free aminopolycarboxylates. Further, key amino acid residues involved in the binding were identified. Our results suggest that EppA is a general binding protein for the uptake of free aminopolycarboxylates. This finding suggests that bacterial cells import free aminopolycarboxylates, explaining why stable metal–chelate complexes are resistant to degradation, as they are not transported into the cells for degradation.

## 1. Introduction

Aminopolycarboxylate chelators (APCs) are amine-containing polycarboxylic acids that are used as metal chelators. Ethylenediaminetetraacetic acid, better known as EDTA, is the most widely used chelator in science, industry, medicine, and consumer goods due to its ability to chelate metals to form stable, water-soluble metal–chelate complexes [1,2]. The stability of metal–EDTA complexes leads to EDTA’s persistence and accumulation in the environment, making it a significant anthropogenic pollutant [3,4,5]. Concerns about EDTA’s potential to mobilize heavy metals, and radionuclides in particular, have led many countries to regulate its use [2,6,7]. Besides EDTA, similar APCs with more specialized applications exist. Both 1,2-bis(2-aminophenoxy)ethane-*N,N,N′,N′*-tetraacetic acid (BAPTA) and ethylene glycol-bis(β-aminoethylether)-*N,N,N′,N′*-tetraacetic acid (EGTA) are used as selective calcium chelators; ethylenediamine-*N,N′*-bis(2-hydroxyphenylacetic acid) (EDDHA) is used to increase the bioavailability of iron for plant fertilization; diethylenetriaminepentaacetic acid (DTPA) is used in numerous applications, such as a contrasting agent in magnetic resonance imaging; *N*-(2-hydroxyethyl)ethylenediamine-*N,N′,N′*-triacetic acid (HEDTA) is used as an iron-based herbicide; and nitrilotriacetate (NTA) is often used in laundry detergents. Their effluence into water supplies may also contribute to persistent APC pollutants. Environmentally friendlier alternatives like the naturally occurring EDDS ((S,S)-ethylenediamine-*N*,*N′*-disuccinic acid) exist [8], and others are under development [9], but the effectiveness and affordability of EDTA have so far ensured its continued use.

One promising method for removing environmental EDTA contamination is bioremediation. For this purpose, multiple bacterial species have been identified that can subsist on EDTA as a sole source of carbon, nitrogen, and energy [10,11,12,13,14]. Being related, the EDTA-degrading bacteria were assigned to the novel genus *Chelativorans* [15]. In *Chelativorans* species, the genes encoding an ATP-binding cassette (ABC)-type transporter system for EDTA uptake and EDTA-degrading enzymes are co-located in a single operon [16]. *Chelativorans* sp. BNC1 (formerly *Mezorhizobium* sp. BNC1), isolated from industrial sewage [17], uses the ABC transport system to uptake EDTA. Inside the cell, EDTA is catabolized [18]. In the first step, an FMNH_2_-dependent EDTA monooxygenase, EmoA, together with its partner NADH:FMN oxidoreductase, EmoB, oxidizes EDTA to ethylenediamine-*N,N′*-diacetate (EDDA) [16,19,20]. Next, iminodiacetate oxidase (IdaA) oxidizes EDDA to ethylenediamine (ED) [21,22]. EmoA and EmoB also oxidize nitrilotriacetate (NTA) to iminodiacetate, and IdaA oxidizes the latter to glycine [16,21]. Each oxidative cleavage produces a glyoxylate molecule, which is used as a carbon source, and the ethylenediamine can be used as a nitrogen source [15].

The EDTA transporter system is composed of a periplasmic binding protein, EppA, and a type I ABC-type importer consisting of a heterodimer of its transmembrane domain components, EppB and EppC, and a dimer of its nucleotide binding domain component, EppD. By sequence comparison, EppA belongs to the periplasmic binding protein PBP2 NikA/DppA/OppA-like superfamily, which is a family in the Class II Cluster C PBP [18]. Class II Cluster C PBPs contain two large polypeptide lobes connected via flexible tethers, allowing them to undergo a large and reversible conformational change, known as the “Venus fly-trap’’ model, in which ligand binding in the cleft between the two lobes induces a closed conformation [23,24]. EppA binds free EDTA, but not metal–EDTA complexes, restricting the ability of *Chelativorans* sp. BNC1 to use only weak metal–EDTA complexes that can dissociate to free EDTA [18], making it imperative to determine the biophysical mechanism of EppA’s binding specificity before any improvements to its binding capabilities can be engineered. We have been delineating the underlying substrate specificity, catalytic mechanism and molecular interactions of key metabolic enzymes—EmoA [20], EmoB [19], and IdaA [22]—in the EDTA-degradation pathway of *Chelativorans* sp. [16]. To understand the first step of EDTA catabolism by *Chelativorans* sp. BNC1 and how it may act as a gatekeeper for all enzymes downstream, here we report structural characterization of EppA and thermodynamic characterization of its binding of EDTA and other APCs.

## 2. Results

### 2.1. Structure of EppA

**Figure 1:**
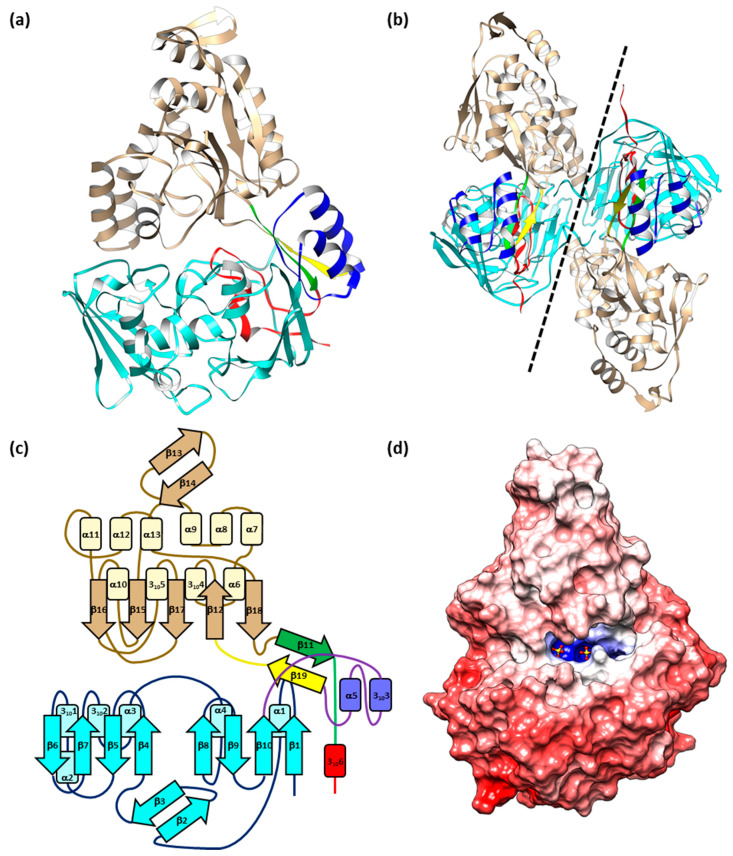
(**a**) Schematic representation of EppA’s structure. Arrow and rectangles are helices and β-strands, respectively. Domain I is aquamarine, Domain IIa is blue, Domain IIb is red, and Domain III is tan. The yellow and green strands, which belong to Domain IIa and IIb, respectively, form the hinge. (**b**) Structure of EppA in the P2_1_2_1_2 space group. The dashed line shows the intermolecular interface between the two monomers, which are related by non-crystallographic 2-fold symmetry. (**c**) Topology diagram of EppA. (**d**) EppA’s electrostatic potential surface on a scale of −12.5 kT/e (red) to +12.5 kT/e (blue), with white at 0 kT/e. The two sulfates in the ligand-binding site are shown as red (oxygen) and yellow (sulfur) ball-and-stick models.

EppA crystallized without its ligand EDTA in the tetragonal space group P4_3_2_1_2 with one molecule in the asymmetric unit (Figure 1a). Soaking the crystals with EDTA lowered their symmetry from P4_3_2_1_2 to P2_1_2_1_2_1_, thereby doubling the molecules in the asymmetric unit to two (Figure 1b); however, no EDTA molecules could be placed unambiguously into the orthorhombic structure after refining the model with EDTA in the ligand-binding cleft. Instead, two sulfate ions from the ammonium sulfate in the crystallization solution were present in the cleft of EppA. For both structures, all but the first four N-terminal residues (Gln27 through Leu30) of the mature protein (Gln27 through Glu563) were visible, and the C-terminal His-tag from pET30a(+) was visible through its first three His residues.

**Figure 2:**
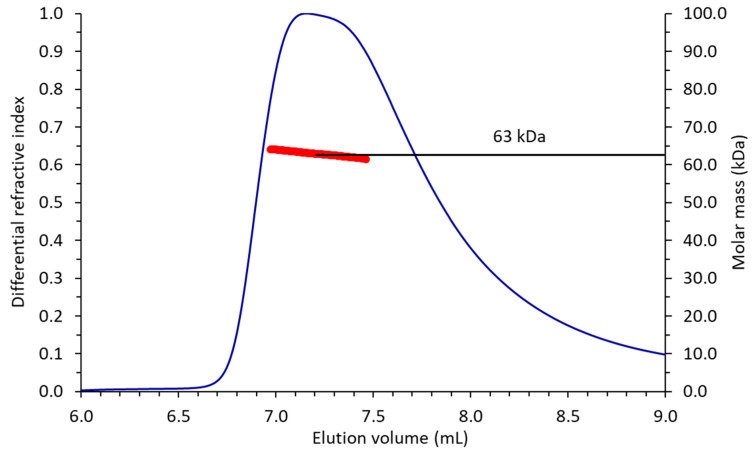
Size-exclusion chromatography–multiangle light scattering profile of EppA. The blue trace is the differential refractive index and the red markers are the calculated molar mass (kDa) at that given elution volume. EppA was monomeric, with an average mass of 63 kDa.

Based on the relatively weak (e.g., no large, hydrophobic intermolecular interfaces) crystallographic and non-crystallographic protein–protein interactions in both the P4_3_2_1_2 and P2_1_2_1_2_1_ crystal lattices, EppA is monomeric, which we confirmed by calculating a molecular mass of 63 kDa for the single chromatographic peak observed by analytical size-exclusion chromatography (Figure 2).

The tertiary structure of EppA was bilobate and composed of three domains, denoted here by convention used for periplasmic binding proteins as Domains I, II, and III. Domain I (Gln27 through Ile256) and Domain III (Ser303 through Leu530) were contiguous domains, whereas Domain II, the smallest of the three, was split into two non-contiguous parts: Domain IIa (Ile257 through Pro302) and Domain IIb (Pro531 to the C-terminus at Glu563). The last eight residues of Domain IIa (Gly295 to Pro302) and first eight residues of Domain IIb (Pro531 to Glu538) each formed a β-strand in an anti-parallel fashion, making a two-strand hinge that links together EppA’s two lobes (Figure 1a). This two-strand hinge, along with the β_2_β_1_β_3_β_n_β_4_ structure of Domain III’s core β-sheet (Figure 1b), classified EppA as a Class II Cluster C periplasmic binding protein [25,26]. Domain I had a structural motif similar to Domain III, consisting of two β -sheets surrounded by loops, α-helices, and a smaller two-strand β sheet between the first and second sheet (Figure 1b). Translation-libration-screw (TLS) [27] analysis of EppA’s structure supported the domain assignments by showing that a two-group partition contained Domains I and IIb for the first group and Domains III and IIb for the second group. Increasing the number of TLS groups partitioned Domain III/IIb further and eventually began to partition Domain IIa.

Through the comparison to other Class II Cluster C PBPs and the support by molecular docking of EDTA (Section 2.3), the interface of Domains I and III established the putative ligand-binding cleft, with one end of the cleft capped by Domain II. In its observed state, this binding cleft had a volume of 688 Å^3^ and was solvent accessible, as shown by the presence of 35 water molecules occupying the extended binding cleft. The cleft was hydrophilic and electrostatically positive (Figure 1d), being lined by the sidechains of Thr55, Arg56, Asn69, Asn70, Ala71, Val72, Arg74, Asn152, and Tyr155 from Domain I; Gln278 and Gln549 from Domain IIa and IIb, respectively; and Tyr415 Asn459, Tyr460, Phe461, Ser462, Gln464, Lys470, Arg480, Gln481, and Tyr483 from Domain III. Domain I and Domain III each contributed two of the four cationic sidechains, whose total 4+ formal charge counterbalances that of EDTA^−4^. Highlighting the counterbalancing charges, electron densities for two sulfate ions were clearly observed to be bound electrostatically in the ligand-binding cleft by Arg56 (water-mediated), Arg74, Lys470, and Arg480. Arg143 bound a third sulfate ion near the ligand-binding cleft. Of the four cationic residues identified by molecular docking, Arg480 was the only residue situated outside of binding cleft within a short loop (Arg477 through Gln481). The loop had two conformers: Conformer A had a refined occupancy of 60% and was held in place outside of the binding clef by Asp447, and Conformer B had a refined occupancy of 40% and was oriented toward the binding cleft.

**Figure 3:**
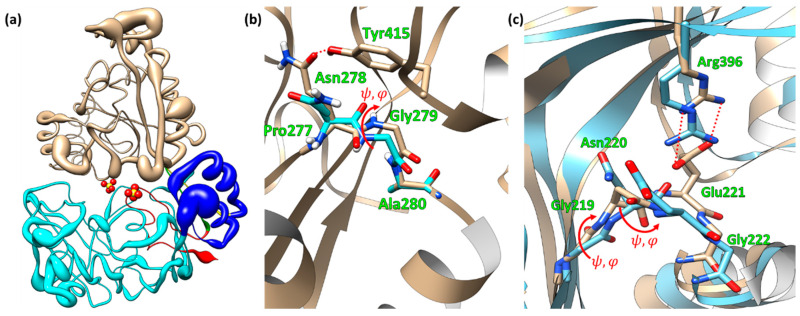
(**a**) EppA’s structure shown with its atomic displacement (average per residue) putty radius. Domain I is cyan, Domain IIa is blue, Domain III is tan, Domain IIb is red, and the hinge are the green and yellow strands. The red and yellow spheres are the two sulfates in the ligand-binding site. Putty radii range from a radius of 0.1 for ADPs at or below 15 Å^2^ to a radius of 3 at 75 Å^2^. (**b**) Alternate conformers of Asn278 and Gly279 in helix-8 of EppA. The cyan sticks are the carbon atoms of the B conformer. (**c**) Comparison of the open (1UIU) and closed (1UIV) forms of NikA at a loop homologous by secondary structure alignment by using COOT to the loop containing Asn278 and Gly279 in EppA.

The EppA structure, depicted via its atomic displacement putty radius (Figure 3a), has three important regions with high isotropic atomic displacements: Domain IIa and two sections of Domain III, one of which is proximal to Domain IIa, the second high displacements region of Domain III being distal to Domain IIb, but connected to the proximal section by helix-15. Notable in Domain IIa was helix-8, a long 3_10_ helix (Gln282 through Leu294), which was capped on both ends by glycine residues (Gly279 and Gly295) and had interacted with the hinge via van der Waals forces (by inspection). Importantly, a short loop before helix-8 had two residues, Asn278 and Gly279, which showed alternate conformers in the electron density map. Asn278 had two alternate sidechain conformers, one with its sidechain pointing out of the ligand-binding site, and its second conformer pointing into the ligand-binding site within hydrogen bonding distance of Tyr415, which itself was held in place by two proline residues (Pro 412 and Pro413) and Tyr460. The φ and Ψ angles of the peptide bond linking Asn278 and Gly279 rotated by 179° and 50°, respectively, between the two conformers (Figure 3b). This suggests that the sidechain conformation of Asn278 is dependent on ligand binding, and by switching conformers, it flips its peptide bond with Gly279, thereby rotating helix-8 in a mechanism analogous to that in NikA from *Escherichia coli* (PDB IDs 1UIV and 1UIU) (Figure 3c) [28].

### 2.2. Isothermal Titration Calorimetry

**Figure 4:**
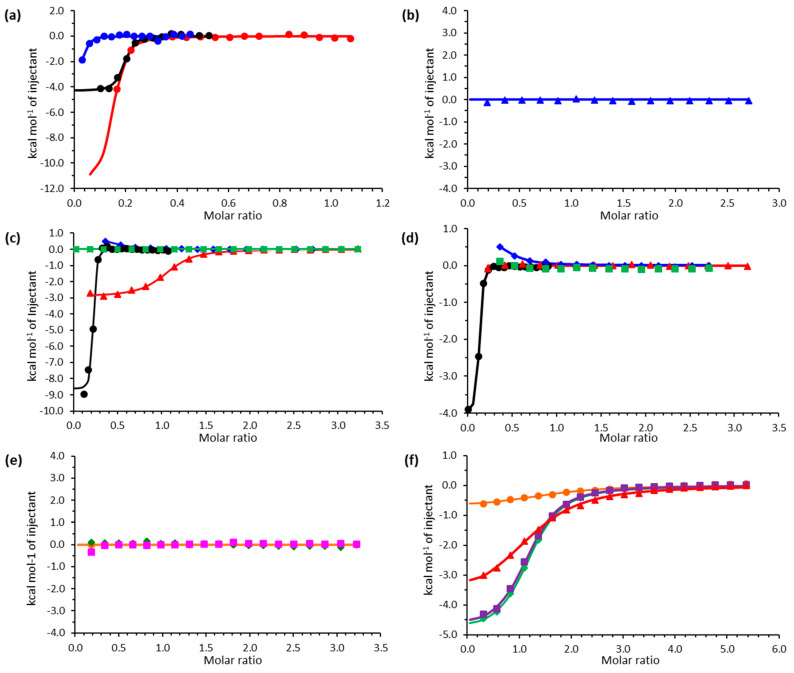
Isothermal titration calorimetry of EppA with various ligands: (**a**) ethylenediamine-*N,N′*-diacetate (EDDA) (blue), ethylenediaminetetraacetate (EDTA) (red), and ethylene glycol-bis(β-aminoethylether)-*N,N,N′,N′*-tetraacetate (EGTA) (black); (**b**) poly-aspartate (decapeptide); (**c**) MgEDTA (black circles), CaEDTA (red triangles), SrEDTA (blue diamonds), and BaEDTA (green squares); (**d**) MgEGTA (black circles), CaEGTA (red triangles), SrEGTA (blue diamonds), and BaEGTA (green squares); (**e**) Co(II)EDTA (green triangles), Mn(II)EDTA (magenta squares), and Fe(III)EDTA (orange circles); (**f**) PrEDTA (green diamond), NdEDTA (purple squares), EuEDTA (red triangles), and TbEDTA (orange circles).

It was previously reported that EppA binds free EDTA [18]; a further investigation of EppA’s ligand-binding abilities was performed by using isothermal titration calorimetry (ITC) to analyze a wider range of metal chelates (Figure 4). Results showed that EppA binds free EDTA and EGTA, with dissociation constants (k_d_) of 9.52 nM and 169 nM, respectively (Table 1). Both ligands bound exothermically and with an increase in entropy, implying that ligand binding and/or closure of the ligand-binding site upon ligand binding is accompanied by solvent/solute release. Binding of EDTA by EppA was more favorable than the binding of EGTA entropically (29 vs. 16.40 cal mol^−1^ K^−1^), but less favorable enthalpically (−2.283 vs. −4.340 kcal mol^−1^). EppA also bound EDDA, albeit weakly (k_d_ = 0.588 μM), but it did not bind decameric polyaspartate (Asp10) (Table 1), suggesting that it did not bind anionic oligopeptides.

EppA bound Mg^2+^–, Ca^2+^–, Sr^2+^–, and Cr^3+^–EDTA chelates with k_d_ of 0.315, 2.32, 1.69, and 0.019 μM, respectively (Table 1). On the other hand, EppA bound only Mg^2+^– and Sr^2+^–EGTA chelates with k_d_ of 0.315 and 4.50 μM, respectively (Table 1). These results were consistent with our previous report that metal–EDTA dissociates first and EppA binds only the dissociated free EDTA [18]. Thus, EppA bound EDTA from CaEDTA^2−^, but not EGTA from CaEGTA^2−^, as CaEGTA^2−^ is more stable than CaEDTA^2−^ [29].

EppA did not bind MnEDTA^2−^, FeEDTA^2−^, CoEDTA^2−^, NiEDTA^2−^, CuEDTA^2−^, ZnEDTA^2−^, and CdEDTA^2−^, suggesting that EppA cannot strip away tightly bound transition metals from EDTA. EppA bound EDTA from Pr^3+^, Nd^3+^, Eu^3+^, and Tb^3+^ chelates, although the obtained k_d_ values decreased with an increasing atomic number of the chelated lanthanide. Complete thermodynamic parameters for all titrations are listed in Table 1.

### 2.3. Molecular Docking

To find the location and conformation of EDTA, EDDA, and EGTA when bound by EppA, and to determine whether EppA bound other aminopolycarboxylates, the ligands were docked into EppA’s structure by using AutoDock Vina [30]. To measure the significance of Arg480′s two alternate conformers on ligand binding, docking was performed for both alternate conformers. The best docked pose of each ligand was chosen with similarity to EDTA and charge neutralization criteria in mind, along with the free energy of binding (ΔG_b_) rankings for both conformers of Arg480 (ΔG_b,A_ and ΔG_b,B_) (Table 2). In most cases, the binding was more favorable with the B conformer.

**Figure 5:**
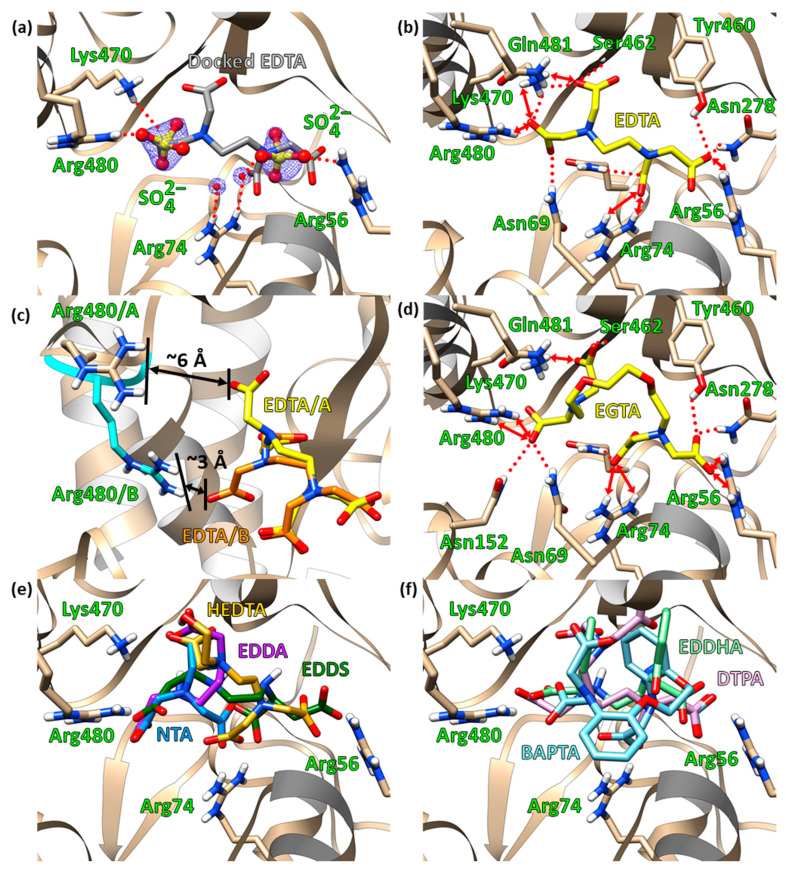
Ligand-binding pocket of EppA. In (**a**), (**b**), and (**d**), hydrogen bonds are depicted by red dotted lines and salt-bridging interactions are depicted by red double-headed arrows; these intermolecular interactions are omitted for clarity in panels (**c**), (**d**), and (**f**). EDTA, EGTA, and residues responsible for these interactions are labeled and shown as sticks; the polypeptide backbone is shown as a ribbon diagram. For all panels, tan (for the protein; ligands are otherwise specified) sticks are carbons, red are oxygens, blue are nitrogens, and white are hydrogens. (**a**) Docked EDTA (gray) superposed onto crystallographic sulfates (yellow and red ball and sticks) that were identified in the F_o_-F_c_ maps of both the tetragonal and orthorhombic structures. The sulfates are shown with their corresponding electron density (blue mesh) contoured at 1.5σ. (**b**) EDTA (yellow) docked into EppA’s ligand-binding cleft. (**c**) Comparison of EDTA docked into the binding pocket of EppA with the loop containing Arg480 in one of its two conformers. The distances show that in Conformer A, hydrogen bond formation is not possible and electrostatics are weak, whereas Conformer B has more favorable electrostatics and is closer to being within range for hydrogen bonding. (**d**) EGTA (yellow) docked. (**e**) Superposition of docked EDDA (purple), EDDS (forest green), HEDTA (gold), and NTA (sky blue). (**f**) Superposition of docked BPTA (pastel blue), DTPA (magenta), and EDDHA (lime green).

As expected, most of the top EDTA conformers bound in the putative ligand-binding cleft. Of EDTA’s four carboxylate arms, two carboxylates were docked at the same position as the two sulfate ions whose total −4 charge neutralizes the four cationic residues—Arg56, Arg74, Lys470, and Arg480—in EppA’s ligand-binding cleft (Figure 5a), giving weight to the docking results. In particular, when docked into EppA with Arg480 in Conformer A, the carboxyl groups on one end of EDTA were positioned near Arg56 and Arg74, and on the other end, one carboxyl group was positioned near Lys470, while the fourth arm was free, giving a ∆G_b_ of −6.4 kcal mol^−1^. When docked into EppA with Arg480 in Conformer B (Figure 5b), each of the carboxyl arms was positioned near one of the four cationic residues, which increased the magnitude of ∆G_b_ to −6.7 kcal mol^−1^, indicating that Conformer B of Arg480 is important for binding (Figure 5c). Accompanying the ionic binding in both cases, Asn69′s Nδ and Tyr460′s phenol were within hydrogen bonding distance of one of EDTA’s carboxylate arms, and the backbone amide of Ser462 was within hydrogen bonding distance of another carboxylate arm.

EGTA, which is larger than EDTA but otherwise has four carboxyl groups like EDTA and was shown by ITC to bind to EppA, docked with a ∆G_b_ of −6.0 kcal mol^−1^ for Conformer A and −6.1 kcal mol^−1^ for the secondary (Figure 5d). Unlike EDTA, EGTA’s best conformer interacted ionically with only Lys470 and Arg480 due to steric clashing from EGTA’s larger size. Other stabilizing interactions were hydrogen bonding with Asn69 and Gln481.

NTA, because of its small size and having only three carboxylates, interacted with only two of EppA’s four cationic sidechains, either Arg56 and Arg74 (∆G_b_ of −5.2 kcal mol^−1^) or Arg74 and Lys470 (∆G_b_ of −5.0 kcal mol^−1^), at once when R480 was in its primary conformer. When docked into EppA with Arg480 in its secondary conformer (Figure 5e), NTA interacted with Arg 74, Lys470, and R480, increasing binding to ∆G_b_ of −6.5 kcal mol^−1^. EDDA, despite being longer than NTA, could only interact with two of the four cationic residues due to having only two carboxylate arms (Figure 5e), binding with a weak ΔG_b_ of −5.2 kcal mol^−1^ for Conformer A and even weaker −4.9 kcal mol^−1^ for Conformer B, making it unlikely that EDDA can close the ligand-binding site. EDDS docked similarly to EDTA (Figure 5e) with a ∆G_b_ of −6.1 kcal mol^−1^ for Conformer A of R480 and −6.7 kcal mol^−1^ for Conformer B. HEDTA, similar to EDTA in size but having one carboxylate replaced by a hydroxymethyl group, interacted with Arg56, Arg74, and Lys470 with ∆G_b_ of −6.1 kcal mol^−1^ when Arg480 was in Conformer A (Figure 5e). When Arg480 was in Conformer B, HEDTA’s three carboxylates were positioned near either Arg74, Lys470, and Arg480 or Arg56, Arg74, and Lys470 with the same ∆G_b_ of −6.1 kcal mol^−1^.

BAPTA, the largest chelator analyzed here, was able to interact with all four cationic residues (Figure 5f) with a ∆G_b_ of −6.9 kcal mol^−1^ for both of Arg480′s conformeric states. DTPA, having five carboxylates and being larger in size than EGTA, bound with a ∆G_b_ of −6.6 kcal mol^−1^ for Conformer A of R480 and −6.7 kcal mol^−1^ for Conformer B (Figure 5f). EDDHA, similar to EDTA but larger and less flexible because one carboxylate on each end is replaced by a phenolate, interacted with Arg74 through a carboxylate on one end and Lys470 and Arg480 with the carboxylate on its other end in both of Arg480′s conformeric states (Figure 5f) with the same ∆G_b_ of −7.0 kcal mol^−1^. The phenolate closest to Lys470 and Arg480 was within hydrogen bonding distance to the backbone amide of Ser462, while the second phenolate was pointed toward solvent.

Despite the more favorable ∆G_b_ of some of the larger chelators, their larger size may inhibit closure of the ligand-binding site. Since molecular docking uses a relatively low level of theory, does not take into account the entropic effects of solvent/solute displacement by ligand binding, and does not accurately model the closure of the ligand-binding cleft, the ∆G_b_ rankings of the chelators were approximations of ligand binding that neglected the full binding mechanism.

### 2.4. The Electrostatic Potentials of the Ligand-Binding Pocket

**Figure 6:**
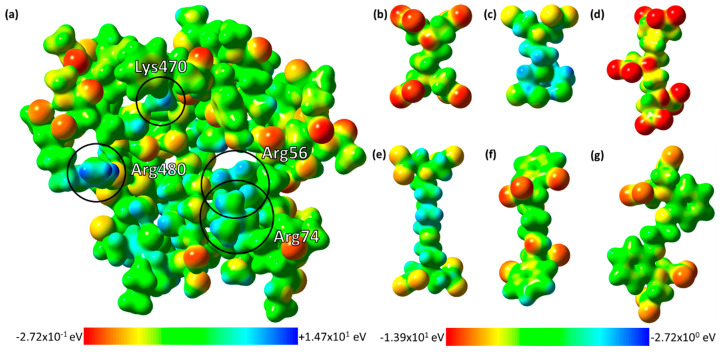
Electrostatic potential surfaces generated from an electronic structure calculation on a model of EppA’s binding pocket (more info in Section 4.8). (**a**) The electrostatic potential of EppA’s ligand-binding pocket mapped onto its electron density at an isovalue of 0.02 electrons bohr^−3^. The potential ranges on a color scale from red (−0.272 eV) to blue (+14.7 eV). Important ligand-binding residues are circled and labeled. The electrostatic potential surfaces of (**b**) EDTA, (**c**) HEDTA, (**d**) DTPA, (**e**) EGTA, (**f**) EDDHA, and (**g**) BAPTA at an isovalue of 0.02 electrons bohr^−3^. The potential ranges on a color scale from red (−13.9 eV) to blue (−2.72 eV).

Electrostatic potential surfaces generated for EppA by the classical Adaptive Poisson–Boltzmann Solver (APBS) method [31] show that EppA’s ligand-binding pocket has a relatively positive electrostatic potential throughout, making it difficult to infer a specific, electrostatics-dependent binding mechanism. Using an electronic structure approach to electrostatic potential surfaces, an alternative approach in this situation, shows that on a quantum chemical level, EppA’s ligand-binding pocket is relatively neutral, but spotted with positive electrostatic potential at Arg56, Arg74, Lys470, and Arg480 (Figure 6a). All the APC ligands in their deprotonated forms had negative electrostatic potential surfaces (Figure 6b–g). The configuration of Arg56, Arg74, Lys470, and Arg480 binding the extended form of EDTA in our docking simulations explains why EppA only binds free EDTA.

### 2.5. Site-Directed Mutagenetic Analysis of Key Binding Residues

To test our hypothesized key EDTA-binding residues, binding of EDTA by EppA with alanine mutants of Arg56 (R56A), Arg74 (R74A), Lys470 (K470A), or Arg480 (R480A) were analyzed by isothermal titration calorimetry (Table 3). R56A, R74A, K470A, and R480A bound EDTA with Kds of 161, 249, 27.1, and 40.8 nM, respectively, which, when compared to 9.52 nM for wild-type EppA, suggests that the cationic residues are important for binding EDTA. Like wild-type EppA, EDTA binding by all four mutants was exothermic (−1.802, −1.877, −1.872, and −1.138 kcal mol^−1^, respectively) and entropically favored (25, 23.9, 28.3, and 30 cal mol^−1^ K^−1^, respectively) (Table 3).

### 2.6. Structural Homologs of EppA and Evolutionary Conservation

To identify homologs of EppA and correlate their sequences with known ligand specificities, the amino acid sequence of EppA was used to perform a similarity search with the deposited crystal structures in the Protein Data Bank (PDB) using position-specific iterative BLAST (PSI-BLAST) [32]. EppA showed low sequence identities to other PBPs (Table 4), and it did not align well with them (Figure S3). Among the ten highest identities, the highest was only 22.7% for LpqW (PDB: 2GRV) from *Mycobacterium smegmatis*, while the lowest was only 17.9% for a chitin oligosaccharide binding protein (CosBP) (PDB: IZTY) from *Vibrio cholerae*. Sequence similarities were higher than identities (38.8% for LpqW and 33.3% for CosBP), but were relatively flat at ~33% for most periplasmic binding proteins (Table 4). The structural homology by pairwise secondary structure superposition of top 10 PSI-BLAST results with EppA were given in Table S1.

Ranking homology by DALI [33] Z-scores (Table 5) showed that the most similar 3D structure was NikZ from *Campylobacter jejuni* (4OET) and AppA from *Bacillus subtilis* (1XOC), both having Z-scores of 34.4 (Table 4). Comparing the Z-scores to their respective identity and similarity from PSI-BLAST (Figure S4) again shows not much correlation between sequence homology and 3D structure. The disconnect could be in part due to the difference between a given PBP’s open and closed forms. Secondary structure superposition of the open and closed forms of NikZ (4OET and 4OES, respectively) showed that the open form of NikZ deviated less from that of EppA than its closed form did (average r.m.s.d of 2.29 Å for both open form chains vs. 2.68 Å for the closed form); however, this trend was reversed in OppA from *Lactococcus lactis*, whose open form (3FTO) had a higher r.m.s.d from EppA of 3.39 Å than its closed form (3DRF), which had an r.m.s.d from EppA of 3.03 Å (full r.m.s.d analysis can be found in Tables S1 and S2). The low sequence identity, but relatively high similarity in 3D structures suggest a common evolutionary history, as they all belong to the Class II Cluster C PBP NikA/DppA/OppA-like superfamily. The structural homology by pairwise secondary structure superposition of top 10 DALI results with EppA were given in Table S2.

Of the four cationic residues identified by molecular docking as being important for ligand binding, Arg56 was identified by ConSurf [34,35,36,37,38] as being a highly conserved residue with a normalized conservation score of −1.019 and a conservation binning of 8, showing preference for only R or K in homologous structures. Arg74, Lys470, and Arg480 were highly variable, having respective conservation scores of 1.255, 0.861, 0.917, and conservation binning of 1, 2, and 2, respectively (Table S3). While the equivalent position of Arg56 in homologous structures preferred only Arg or Lys, Arg 74, Lys470, and Arg480 preferred small residues (Ala, Gly, Ser), amides (Asn and Gln), and charged residues (Asp, Glu, Arg, and Lys). These analyses indicated that EppA’s ability to bind EDTA has evolved primarily through adopting Arg74, Lys470, and Arg480, while Arg56 was likely adventitious.

## 3. Discussion

**Figure 7:**
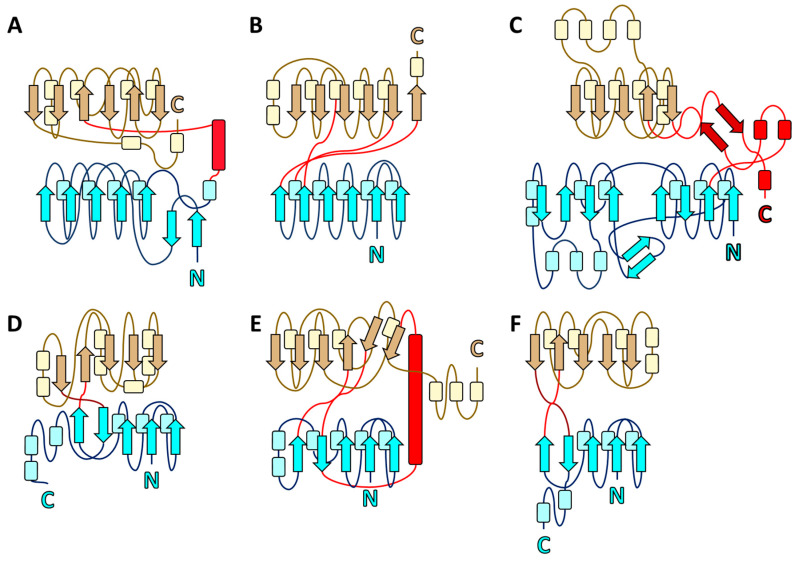
Topology diagrams of representative proteins of the Class II PBPs. For each diagram, secondary structural elements (not to scale) are arrows for β strands, cylinders for helices (all types), and lines for loops. Secondary structural elements are colored cyan (strands), light blue (helices), and blue (loops) for Domain I; crimson (strands) and red (loops and helices) for Domain II; and brown (strands and loops) or yellow (helices) for Domain III. Clusters (**A**) (HtsA from *Staphylococcus. aureus*; PDB ID 3EIW), (**B**) (GGBP from *Escherichia coli*; PDB ID 2FVY), (**C**) (OppA from *Salmonella. typhimurium*; PDB ID 1JET), (**D**) (phosphate-binding protein from *E. coli*; PDB ID 11XH), (**E**) (TTHA0776 from *Thermus thermophilus* HB8; PDB ID 2ZZV), and (**F**) (ModA from *Azotobacter vinelandii*; PDB ID 1ATG).

Based on its three-domain, bilobate structure, the β_2_β_1_β_3_β_n_β_4_ configuration of the core β-sheet of Domain III, and the two-β-strand hinge, EppA is a Class II Cluster C periplasmic binding protein [25,39]. For the Clusters A, B, D, E, and F of PBPs, their Domains I and III are relatively symmetric in size and shape, and the two domains of Cluster C PBPs like OppA, NikA and EppA are significantly asymmetric (Figure 7). This asymmetry among the Cluster C PBPs is possibly reflected in the heterodimeric composition of their ABC transporter’s transmembrane region, consisting of two different proteins (EppB and EppC), while the ABC importers associated with other PBP clusters are often homodimeric transmembrane proteins [40].

Due to the high ammonium sulfate concentrations (1 to 2 M) in all of the conditions that EppA crystallized in, sulfate ions occupied EppA’s binding cleft, preventing formation of an EppA–EDTA complex. When 100 mM EDTA was added to the crystals, it caused the crystals to melt. To determine EppA’s EDTA-binding mechanism, we resorted to molecular docking. Molecular docking suggests that EppA binds its ligands by salt bridging interaction between the ligands and the cationic sidechains of Arg56, Arg74, Lys470, and Arg480. The site-directed mutation of R56A, R74A, K470A, and R480A in EppA resulted in a 17-, 26-, 3-, and 4-fold reduction in EDTA binding, respectively (Table 3), suggesting Arg56 and Arg74 are more critical in the substrate binding than Lys470, and Arg480. Further, the electronic structure analysis supports that EppA’s ligand-binding pocket is relatively neutral with positive charged Arg56, Arg74, Lys470, and Arg480 that directly interact with negatively charged carboxylic groups of ETDA (Figure 6a).

The short loop (Arg477 through Gln481) with Arg480 outside of the binding cleft may function as a gate. Conformer A positioned Arg480′s guanidium sidechain too far (~6 Å) from the docked EDTA, and Conformer B positioned Arg480′s sidechain within a reasonable distance for binding (~3 Å) (Figure 5c). The apparent conformeric flexibility of the loop may allow Arg480 to serve as an actuator for closing the ligand-binding cleft, as Arg480 in Conformer B appeared interacting with one of the bound sulfates. Since both cationic sidechains of Arg56 and Arg74 from Domain I and Lys470 from Domain III lie closely within the binding cleft, EDTA likely binds to them first, followed by Arg480 in Domain III when the short loop is in Conformer B, an event that would close the binding site in a manner consistent with the Venus flytrap mechanism, as observed in other periplasmic binding proteins [23,24].

Strong metal–EDTA chelates cannot bind to EppA for two reasons. First, metal–EDTA complexes are spherical, compact molecules that cannot span the gap between Arg56, Arg74, Lys470, and Arg480 in the binding pocket of EppA. Docking simulations show the best docked MgEDTA reaching only 3.67 Å from Lys470, the three other residues being even further away (~5 Å)(Figure S5). Second, there are no apparent means by which EppA can interact with a metal–EDTA complex’s metal center. NikA is the binding protein required for the uptake of Ni^2+^, and it binds FeEDTA(H_2_O), suggesting a natural metallophore is required to complex with Ni^2+^ before its uptake. When binding FeEDTA(H_2_O), NikA uses its Arg97 and Arg137 sidechains to bind one carboxylate each with reasonable intermolecular distances (~2.8 Å), and it interacts with the metal center by means of a π–cation interaction with Trp398 of Domain III [41,42]. By structural superposition, the π–cation interaction appears to be responsible in part for the closure of NikA’s ligand-binding site. No Trp sidechains are within EppA’s binding site, making the existence of a homologous π–cation interaction in EppA unlikely, thereby explaining why it cannot bind strong metal–EDTA complexes.

In summary, EppA’s affinity for EDTA, EDDA, and EGTA, and its putative affinity for other APC chelators via molecular docking, suggests that it is a general binding protein for aminopolycarboxylates. We speculate that EppA’s original function might have been to bind naturally occurring aminopolycarboxylates, such as ethylenediaminedisuccinate [43], considering that EDTA was only first synthesized in 1935 [44]. Binding of ligands by PBPs is a prerequisite for their import to the bacterial cytoplasm by the PBP’s cognate ABC transporter. Of the aminopolycarboxylates, DTPA and NTA are also substrates for EmoA [45], and EDDS is a substrate of both the bacterium BNC1 and a related EDTA-degrading bacterium *Chelativorans multitrophicus* DSM 9103 [8,15]; therefore, EppA may participate in transporting EDTA as well as other aminopolycarboxylates into *Chelativorans* sp. for biodegradation. In the case of weak metal–chelate complexes, EppA likely facilitates dissociation of the weak chelates by using its cationic residues to weaken the carboxylate-mediated metal–chelate bonds and bind the carboxylates, opening up EDTA to its extended conformation and releasing the metal. Since EppA can facilitates the uptake of free synthetic and natural aminopolycarboxylates, the stable metal–chelate complexes will not be subject to EppA-dependent uptake for biodegradation in the cytoplasm, explaining the recalcitrant nature of aminopolycarboxylates in natural environments.

## 4. Materials and Methods

### 4.1. Site-Directed Mutagenesis

R56A, R74A, K470A, and R480A EppA mutants were generated by site-directed mutagenesis of the wild-type EppA gene (GenBank: ABG63228.1) using the standard Phusion protocol. All primers were ordered from Invitrogen (Carlsbad, CA, USA). The stability of the mutant proteins were supported by molecular mechanics optimizations and the fact that their chromatographic elution profiles during purification were consistent with wild-type EppA.

### 4.2. Protein Expression and Purification

BL21(DE3)pLysS *E. coli* cells containing the *eppA* gene inserted into pET−30a(+) Ek/LIC were grown in lysogeny broth at 37 °C and induced by adding 0.5 mM isopropyl β-D-1-thiogalactopyranoside (IPTG) after reaching an A600 of 0.8. Following induction, the cells were harvested, suspended in Ni-NTA wash buffer (50 mM NaPi, 0.5 M NaCl, 20 mM imidazole, 0.05 g dL^−1^ NaN_3_, pH 8.0), and sonicated to apparent homogeneity using a 450 Sonifier^®^ (Branson Ultrasonics; Danbury, CT, USA). The crude lysate was clarified by centrifugation at 41,107× *g* and loaded onto a Ni-NTA (G Biosciences) column equilibrated with wash buffer. After washing the column thoroughly with wash buffer, EppA was eluted from the column with Ni-NTA elution buffer (50 mM NaPi, 0.5 M NaCl, 250 mM imidazole, 0.05 g dL^−1^ NaN_3_, pH 8.0). The Ni-NTA elution fraction was concentrated, buffer exchanged into anion exchange buffer A (20 mM Tris, 0.05 g dL^−1^ NaN_3_, pH 8.5), and injected onto a Mono Q column (Cytiva, Marlborough, MA, USA). EppA eluted between a gradient of 10 to 13% anion exchange buffer B (20 mM Tris, 2.0 M NaCl, 0.05 g dL^−1^ NaN_3_, pH 8.5). Fractions containing EppA were then pooled, concentrated, and dialyzed into EppA assay buffer (20 mM MOPS, 0.15 M NaCl, 0.05 g dL^−1^ NaN_3_, pH 7.2) for ITC, MALS, and crystallization. Protein concentrations were measured by bicinchoninic acid (BCA) assay (Thermo Fisher Scientific; Carlsbad, CA, USA).

### 4.3. Molecular Mass Determination

In total, 200 μg of EppA was injected onto a Yarra 3u SEC-2000 (Phenomenex; Torrance, CA, USA) size-exclusion column and eluted isocratically by EppA assay buffer. The 280 nm absorbance, laser light scattering, and differential refractive index were measured in tandem by a 280 nm UV detector (Agilent Technologies 1260 Infinity II), a DAWN HELEOS II 8+ (Wyatt Technology; Santa Barbara, CA, USA), and an Optilab T-rEX (Wyatt Technology; Santa Barbara, CA, USA), respectively. The molecular mass of EppA was calculated in ASTRA 7.1.4.8 (Wyatt Technology; Santa Barbara, CA, USA) by Zimm fitting measured light scattering intensities.

### 4.4. Crystallization

Initial crystallization trials using sparse matrix screening from Anatrace (Maumee, OH, USA) and Hampton Research (Aliso Viejo, CA, USA) were set up by a Phoenix RE (Art Robbins Instruments; Sunnyvale, CA, USA). The best screening solution was optimized and used for all crystal growth. Crystals were grown using the hanging drop vapor diffusion method with 1.5 μL of EppA (625 μM in 20 mM MOPS, 0.15 M NaCl, 0.05 g dL^−1^ NaN_3_, pH 7.2) mixed with 1.5 μL of mother liquor (0.1 M Tris, 2.0 M (NH_4_)_2_SO_4_, pH 8.0) over a 500 μL reservoir of mother liquor at 4 °C. Crystals finished growing by two months.

### 4.5. Structure Determination

Crystallographic data were collected at the Advanced Light Source (Beamline 5.0.2) and integrated, reduced, and scaled using HKL2000 [46]. The structure of the periplasmic binding protein TM1223 from *Thermotoga maritima* (PDB ID: 1VR5) was used as a template in SWISS-MODEL (Computational Structural Biology Group at the SIB Swiss Institute of Bioinformatics at the Biozentrum, University of Basel; Basel, Basel-City, Switzerland) [47,48,49,50] to generate a homology model of EppA since of all possible templates, TM1223 had the third highest identity via PSI-BLAST of 20.0% and the second highest GMQE score of 0.58 from SWISS-MODEL, making it the best combination of both among all homology models. After deleting all of the homology model’s sidechains, its two core β sheets of Domain I and core β sheet of Domain III were used as an input model along with the P4_3_2_1_2 dataset for molecular replacement in PHENIX (PHENIX Industrial Consortium; Berkeley, CA, USA) [51]. After obtaining initial phases from the core β-sheet model, sections of the sidechain-free homology model were fitted to the electron density where appropriate using COOT (Biomedical Campus, Cambridge, UK) [52] and refined in PHENIX. This process was performed iteratively to build a successively more complete partial model as phases improved until the model was complete enough to be built finished by PHENIX AutoBuild [53], after which the rest of the model was built by hand. Iterative adjustment and refinement of the AutoBuild solution were performed in COOT and PHENIX, respectively. TLS groups were identified by the TLSMD web server [27] after the isotropic atomic displacement parameters had sufficiently converged. Crystallographic coordinates and structure factors have been deposited in the Research Collaboratory for Structural Bioinformatics Protein Data Bank (RCSB PDB) with PDB IDs of 6WM6 for the tetragonal space group and 6WM7 for the orthorhombic space group [54,55]. Refinement statistics are listed in 5.

### 4.6. Structure Analysis

PDB2PQR (Battelle Memorial Institute, Columbus, OH, USA) [56] was used to prepare crystallographic/homology model coordinates for whole-model electrostatic potential surface calculations. Electrostatic potentials were then calculated for the prepared models by APBS (Battelle Memorial Institute, Columbus, OH, USA) [31] and mapped onto their respective solvent-excluded molecular surface using the MSMS package [57] in UCSF Chimera (Resource for Biocomputing, Visualization, and Informatics at University of California, San Francisco; San Francisco, CA, USA) [58]. The binding cleft volume (concerning the solvent excluded surface) was calculated using the CASTp 3.0 web server (University of Illinois at Chicago, Chicago, IL, USA) [59]. Backbone torsion comparisons of the open and closed forms of TM1223 (1VR5 and 4PFT), respectively) were made by extracting torsion angles from their respective structures after refinement against their deposited structure factors to correct for geometric errors. Evolutionary conservation was analyzed by submitting the coordinates of EppA to the ConSurf server (Tel Aviv University, Tel Aviv, IL, USA) [34,35,36,37,38]. For ConSurf, the multiple sequence alignment was built using MAFFT, homologues were collected from the UNIREF90 database by two iterations of PSI-BLAST (E-value: 0.0001), and conservation scores of homologues was calculated using a Bayesian method.

### 4.7. Isothermal Titration Calorimetry

Isothermal calorimetric titrations were performed in a MicroCal iTC200 (Malvern Panalytical Ltd.; Malvern, UK). All titrations were performed at 25 °C and stirred at 750 rpm. Ligand solutions (0.5 mM for EDTA, EGTA, MgEDTA, and MgEGTA; 1.5 mM for CaEDTA, SrEDTA, BaEDTA, and the transition metal EDTA chelates; and 2.5 mM for the lanthanide EDTA chelates) were injected into the calorimetric cell containing 100 μM EppA in assay buffer as either sixteen injections (one 0.8 μL injection followed by fifteen 2.47 μL injections) for free EDTA, free EGTA, and the Mg chelates, or twenty injections (0.8 μL injection followed by nineteen 1.8 μL injections) for all other chelates. EppA–ligand binding curves were corrected for heats of dilution by subtracting reference titrations of ligands at the same concentrations into a buffer without EppA. Data were fitted to a single-site model as implemented into the Origin 7 MicroCal Data Analysis software analysis package (Malvern Panalytical Ltd.; Malvern, UK) and then plotted in Excel.

### 4.8. Quantum Chemical Optimization and Electrostatics

EppA was prepared for quantum mechanical electrostatic potential surface generation by reducing its crystallographic solvent and optimizing its hydrogen bond network using the PDB Prep Wizard and propKa in Schrödinger Maestro (Schrödinger; Portland, OR, USA) [60,61]. The hydrogen atoms of the reduced and hydrogen bonding-optimized model were then optimized using the AMBER molecular force field [62] in Gaussian 09 (Gaussian, Inc.; Wallingford, CT, USA) [63]. A single-point calculation was then performed on a smaller model (Thr55, Arg56, Asp57, Val63, Thr64, Ser65, Ala66, Leu67, Gly68, Asn69, Asn70, Ala71, Val72, Val73, Arg74, Thr75, Ile151, Asn152, Ala153, Ser154, Tyr155, Pro277, Gln278, Gly279, Ala304, Asn305, Trp306, Asn459, Tyr460, Phe461, Ser462, Gln463, Val465, Val469, Lys470, Ala471, Gly472, Gln473, Ile474, Phe475, Thr479, Arg480, Gln481, Asn482, Pro547, Asn548, Gln549, Leu550, and Gly551) at the CAM-B3LYP level of theory [64] using 3-21G basis sets [65,66] for all atoms in Gaussian 09. The 12 point bohr^−1^ total electron density and electrostatic potential grids were then generated from the single-point self-consistent field density by the Gaussian 09 cubegen utility and mapped in GaussView 5.09 (Gaussian, Inc.; Wallingford, CT, USA) [67] as the electrostatic potential on the electron density at an isovalue of 0.02 electrons bohr^−3^.

BAPTA, DTPA, EDDA, EDDHA, EDDS, EDTA, EGTA, HEDTA, and NTA, in their fully ionized forms, were generated from their SMILES codes by Phenix eLBOW [68] and optimized in Gaussian 09 at the CAM-B3LYP level of theory with double-ζ correlation-consistent basis sets (cc-pVDZ) that were augmented for carbon, nitrogen, and oxygen atoms. The lowest energy conformation of each ligand was found by relaxed potential energy surface scans around relevant dihedral angles and was confirmed by frequency analysis of the optimized structure. A single-point calculation was then ran on each optimized structure at the CAM-B3LYP level of theory with triple-ζ correlation-consistent basis sets (cc-pVTZ) that were augmented for carbon, nitrogen, and oxygen atoms [69,70]. Electrostatic potential surfaces were generated using the same method as described for EppA’s ligand-binding cleft.

### 4.9. Molecular Docking

QM-optimized BAPTA, DTPA, EDDA, EDDHA, EDDS, EDTA, EGTA, HEDTA, and NTA were docked into EppA by AutoDock Vina [30]; ligands and grids were prepared for docking using AutoDock Tools [71]. Each ligand was docked into EppA with Arg480 in Conformer A and Conformer B, and each run with EppA set as a rigid receptor. EppA’s EDTA binding site was found by blind docking EDTA into a whole-protein search grid. The lowest energy binding position was then used to center a 15 Å × 20 Å × 15 Å grid at the coordinates (26.372 Å, 53.932 Å, 67.442 Å) into which EDTA was docked again. BAPTA, DTPA, EDDA, EDDHA, EDDS, EGTA, HEDTA, and NTA were docked into the same grid centered on the same coordinates.

## Figures and Tables

**Table 1:** Thermodynamic parameters determined by isothermal titration calorimetry. ^a^

	K_d_ (μM)	∆H (kcal mol^−1^)	∆S (cal mol^−1^ K^−1^)
Asp10 ^b^	n.b. ^c^	n.b.	n.b.
EDDA ^d^	0.340 ± 0.118	−0.851 ± 0.893	26.7
EDTA	0.0095 ± 0.006	−2.283 ± 0.07	29
EGTA	0.169 ± 0.71	−4.34 ± 0.114	16.4
MgEDTA	0.315 ± 3.26	−4.028 ± 0.037	16.2
CaEDTA	2.320 ± 20.41	−2.917 ± 0.038	16.0
SrEDTA	1.69 ± 7.69	−4.392 ± 0.118	27.1
BaEDTA	n.b.	n.b.	n.b.
CrEDTA	0.019 ± 0.002	−2.573 ± 0.145	30.5
MnEDTA	n.b.	n.b.	n.b.
FeEDTA	n.b.	n.b.	n.b.
CoEDTA	n.b.	n.b.	n.b.
NiEDTA	n.b.	n.b.	n.b.
CuEDTA	n.b.	n.b.	n.b.
ZnEDTA	n.b.	n.b.	n.b.
CdEDTA	0.28 ± 0.49	−0.555 ± 0.002	28.1
PrEDTA	6.17 ± 55.25	−5.366 ± 0.133	5.83
NdEDTA	8.85 ± 141.2	−4.889 ± 0.063	6.73
EuEDTA	26.45 ± 363.6	−3.943 ± 0.105	7.72
TbEDTA	31.25 ± 116.4	−0.751 ± 0.069	18.1
MgEGTA	0.315 ± 3.26	−4.028 ± 0.037	16.2
CaEGTA	n.b.	n.b.	n.b.
SrEGTA	4.50 ± 10.5	0.794 ± 0.164	27.1
BaEGTA	n.b.	n.b.	n.b.

^a^ The titrations were performed in single replicate. Representative ITC raw data are given in Figure S1. ^b^ Asp10: decameric polyaspartate. ^c^ n.b.: no binding. ^d^ Abbreviations: EDDA (ethylenediamine-*N,N′*-diacetate), EDTA (Ethylenediaminetetraacetate), EGTA (ethylene glycol-bis(β-aminoethylether)-*N,N,N′,N′*-tetraacetate).

**Table 2:** Binding free energies calculated by molecular docking.

Ligand *	ΔG_binding,A_ (kcal mol^−1^)	ΔG_binding,B_ (kcal mol^−1^)
EDTA	−6.4	−6.6
EGTA	−6.0	−6.1
NTA	−5.2	−6.5
EDDA	−5.2	−4.9
HEDTA	−6.0	−6.1
EDDS	−6.1	−6.7
DTPA	−6.6	−6.7
EDDHA	−7.0	−7.0
BAPTA	−6.9	−6.9

* Abbreviations: NTA (nitrilotriacetate), HEDTA (*N*-(2-hydroxyethyl)ethylenediamine-*N,N′,N′*-triacetate), EDDS ((S,S)-ethylenediamine-*N*,*N′*-disuccinate), DTPA (diethylenetriaminepentaacetate), ethylenediamine-*N,N′*-bis(2-hydroxyphenylacetate), and BAPTA (1,2-bis(2-aminophenoxy)ethane-*N,N,N′,N′*-tetraacetate).

**Table 3:** Thermodynamic parameters for binding of EDTA by site-directed EppA mutants. ^a^

	K_d_ (nM)	∆H (kcal mol^−1^)	∆S (cal mol^−1^ K^−1^)
Wild-type	9.52 ± 6.25	−2.283 ± 0.070	29
R56A	161 ± 272	−1.802 ± 0.147	25
R74A	249 ± 382	−1.877 ± 0.195	23.9
K470A	27.1 ± 20.2	−1.872 ± 0.117	28.3
R480A	40.8 ± 26.5	−1.138 ± 0.089	30

^a^ The titrations were performed in single replicate. Representative ITC raw data are given in Figure S2.

**Table 4:** EppA homology via position-specific iterative (PSI)-BLAST and comparison to Z-scores.

Protein	PDB ID	Source	% Identity	% Similarity	Z-Score
LpqW	2GRV	*Mycobacterium smegmatis*	22.5	38.8	29.0
CBP	2O7I	*Thermotoga maritima*	19.6	33.5	24.4
AgaA	6HLX	*Rhizobium radiobacter*	18.8	35.7	31.4
OppA	3DRF	*Lactococcus lactis*	18.7	35.2	27.6
OppA2	2WOK	*Streptomyces clavuligerus*	18.3	32.3	29.1
MnBP3	4PFT	*Thermotoga maritima*	18.3	35.3	27.6
MoaA	6TFX	*Rhizobium radiobacter*	18.2	33.5	30.3
NikZ	4OET	*Campylobacter jejuni*	18.1	36.8	34.4
CtaP	5ISU	*Listeria monocytogenes*	18.0	33.9	34.2
CosBP	1ZTY	*Vibrio cholerae*	17.9	33.3	21.0

**Table 5:** X-ray diffraction data collection and structure refinement statistics.

	EppA (apo)	EppA (EDTA Soak)
Data collection		
Space group	P4_3_2_1_2	P2_1_2_1_2_1_
Cell dimensions		
a, b, c (Å)	87.81, 87.81, 164.48	84.34, 90.61, 165.27
α, β, γ (°)	90.00, 90.00, 90.00	90.00, 90.00, 90.00
Resolution (Å)	49.46—1.42 (1.47—1.42)	47.07—1.56 (1.62—1.56)
R_merge_	0.16 (0.860)	0.125 (1.59)
Wavelength (Å)	0.9793	1.000
Unique reflections	122,095 (11,723)	179,416 (17,354)
Completeness (%)	97.40 (89.55)	94.44 (77.22)
<I>/σI	10.57 (4.77)	12.65 (1.22)
CC1/2	0.996 (0.887)	0.999 (0.375)
CC *	0.999 (0.97)	1 (0.738)
Multiplicity	20.5 (16.6)	13.5 (6.2)
Refinement		
R_work_/R_free_	0.145 (0.187)/0.153 (0.210)	0.153 (0.310)/0.169 (0.317)
CC_work_/CC_free_	0.971 (0.919)/0.966 (0.915)	0.968 (0.709)/0.966 (0.691)
Number of atoms		
Protein	4209	8310
Sulfate	20	15
Ethylene glycol	63	80
Water	604	1285
ADP (Å^2^)		
All atoms	26.05	21.33
Protein	24.45	19.57
Ligands	34.5	30.2
Solvent	36.58	32.04
R.m.s deviations		
Bonds (Å)	0.008	0.008
Angles (°)	1.31	1.27
Ramachandrans		
% Favored	98.31	98.12
% Outliers	0	0
Rotamer outliers	0.45	0.23
Clashscore	1.79	2.29
TLS groups	3	6

* Parenthetical values are statistics for their respective highest resolution shell.

## Supplementary Materials

The following are available online at https://www.mdpi.com/1422-0067/21/11/3940/s1.
