# Supplementary material for: The Structural Basis of the Binding of Various Aminopolycarboxylates by the Periplasmic EDTA-Binding Protein EppA from Chelativorans sp. BNC1
Source: Int J Mol Sci. 2020 May 30;21(11):3940. doi: 10.3390/ijms21113940 (PMC7312458; doi:10.3390/ijms21113940)
Supplement: Supplementary file 1 [file ijms-21-03940-s001.pdf]

The structural basis of the binding of various aminopolycarboxylates by the periplasmic EDTA-binding protein EppA from *Chelativorans* sp. BNC1

Kevin M. Lewis[1], Chelsie L. Greene[1], Steven A. Sattler[2], Buhyun Youn[3], Luying Xun[2*], and ChulHee Kang[1,2*]

[1] Department of Chemistry, Washington State University, Pullman, Washington, USA; kevin_lewis@wsu.edu (K.L.); chelsie.boro@wsu.edu (C.G.)
[2] School of Molecular Biosciences, Washington State University, Pullman, Washington, USA; s.andrew.sattler@gmail.com (S.S.)
[3] Department of Biological Sciences, College of Natural Sciences, Pusan National University, Busan, Republic of Korea; bhyoun72@pusan.ac.kr (B.Y.)
* Correspondence: luying_xun@wsu.edu (L.X.) and chkang@wsu.edu (C.K.)

Supplemental Figures

Figure S1. Isothermal titration calorimetry data for titration of wild-type EppA with varying chelators and chelates.

Figure S2. Isothermal titration calorimetry data for titration of wild-type EppA and four mutants with EDTA.

Figure S3. CLUSTAL Omega Multiple Sequence Alignment of Top Ten PSI-BLAST Matches.

Figure S4. CLUSTAL Omega Multiple Sequence Alignment of Top Ten DALI Results.

Figure S5. Docking of MgEDTA to EppA.

Table S1. Structural Homology by Pairwise Secondary Structure Superposition of Top Ten PSI-BLAST Results with EppA.

Table S2. Structural Homology by Pairwise Secondary Structure Superposition of Top Ten DALI Results with EppA.

Table S3. ConSurf amino acid conservation scores.

Figure S1. Isothermal titration calorimetry data for titration of wild-type EppA with varying chelators and chelates.

Decameric poly-aspartate and alkaline earth EDTA chelates:

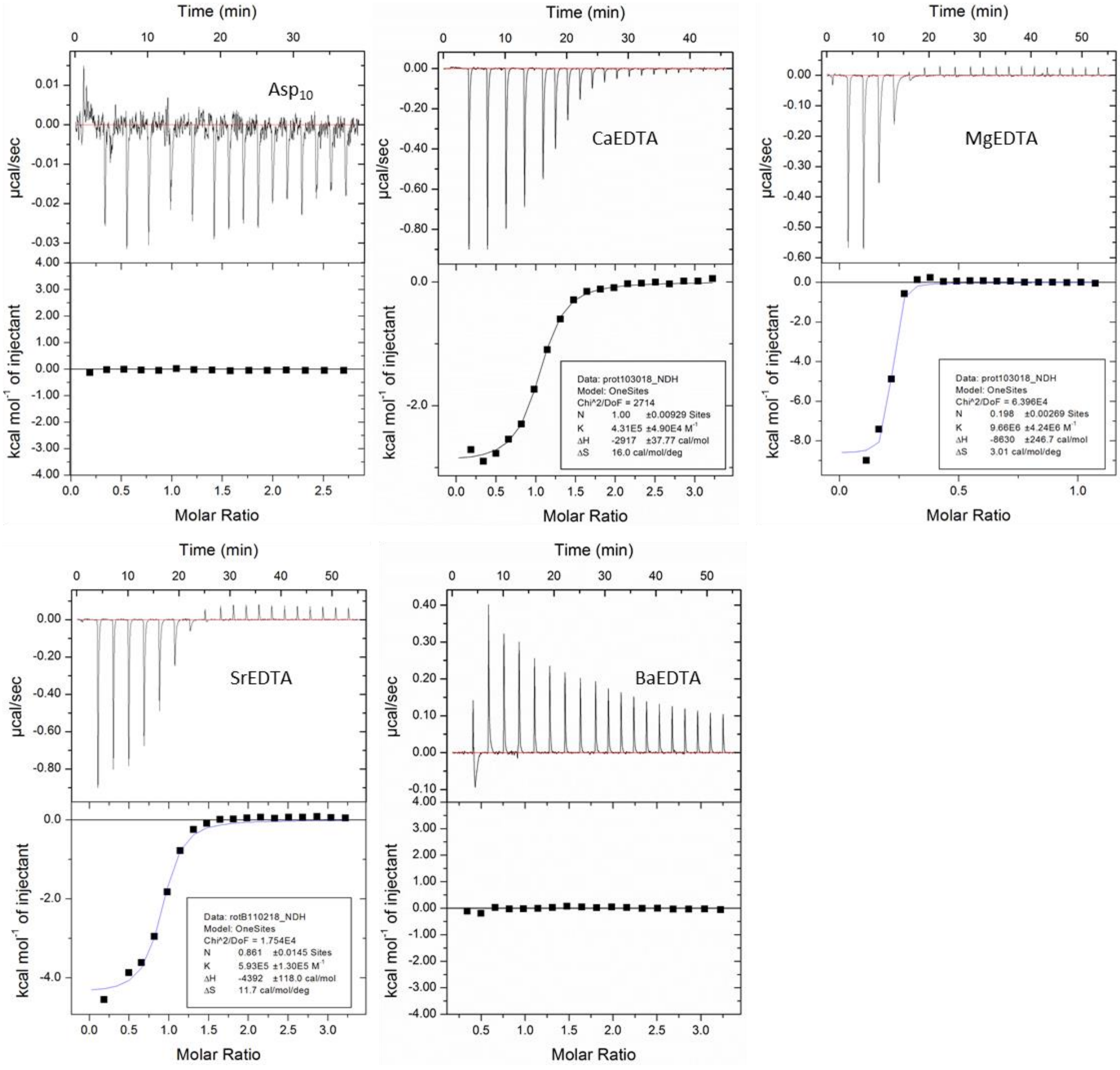

Transition metal chelates:

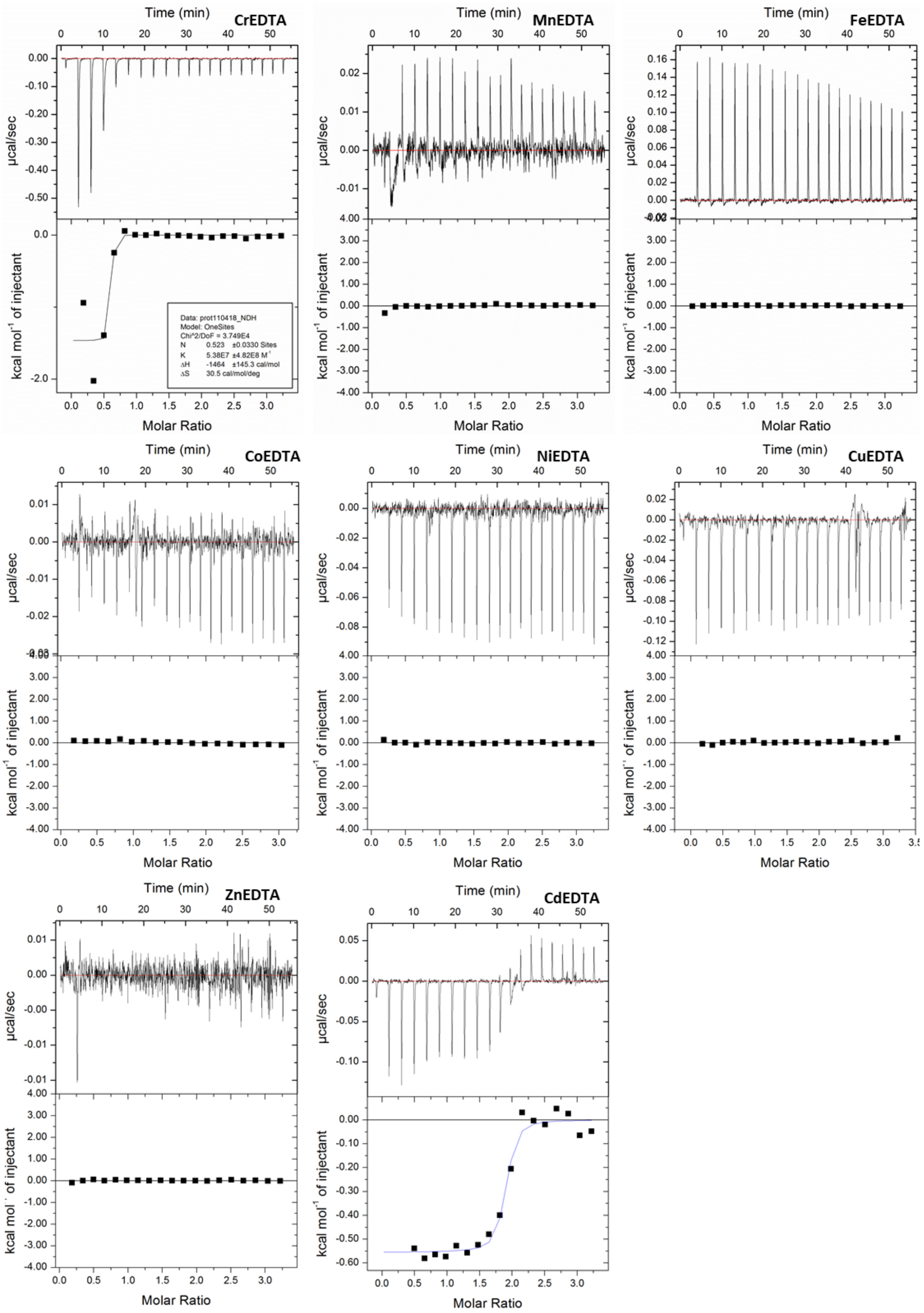

Lanthanide EDTA chelates:

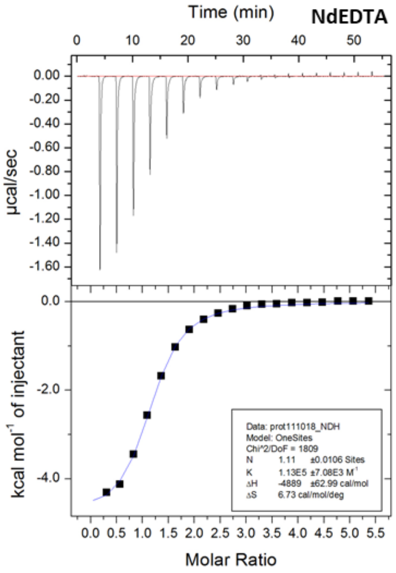

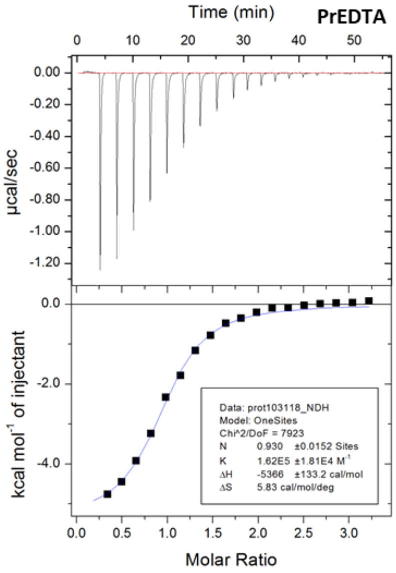

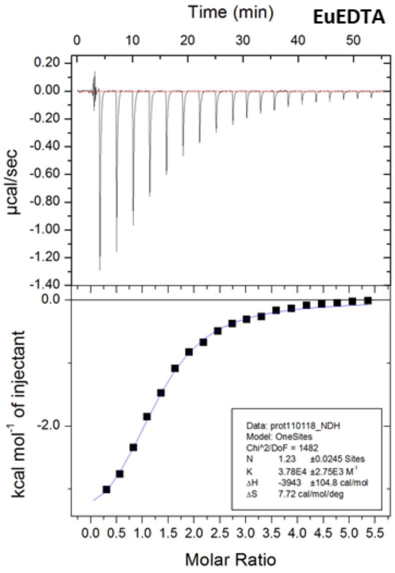

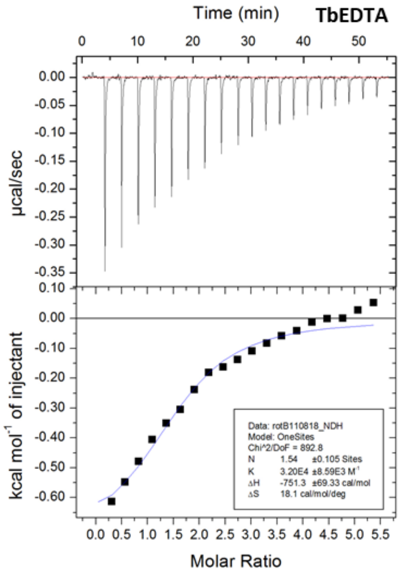

EGTA and alkaline earth EGTA chelates:

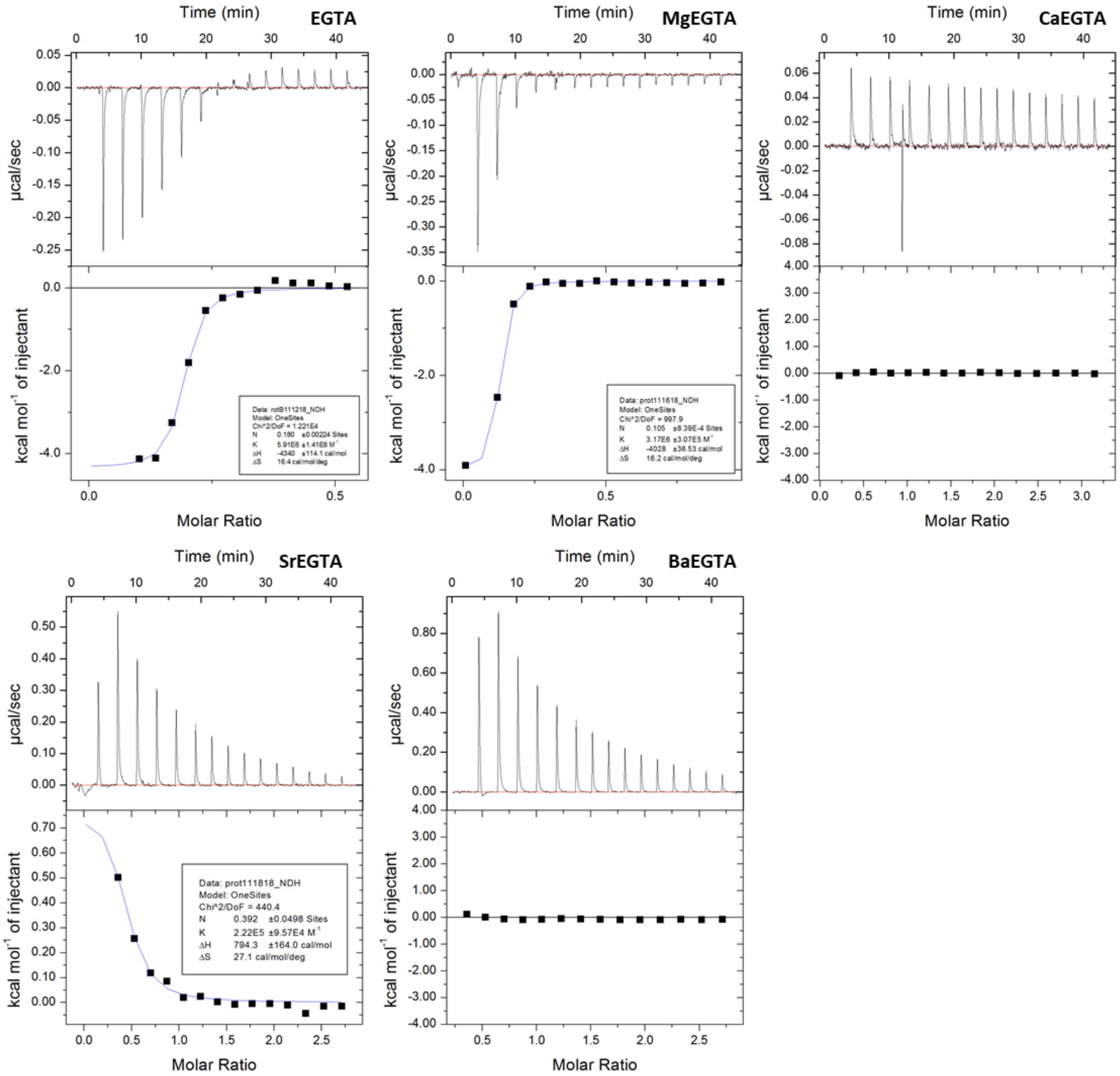

Figure S2. Isothermal titration calorimetry data for titration of wild-type EppA and four mutants with EDTA.

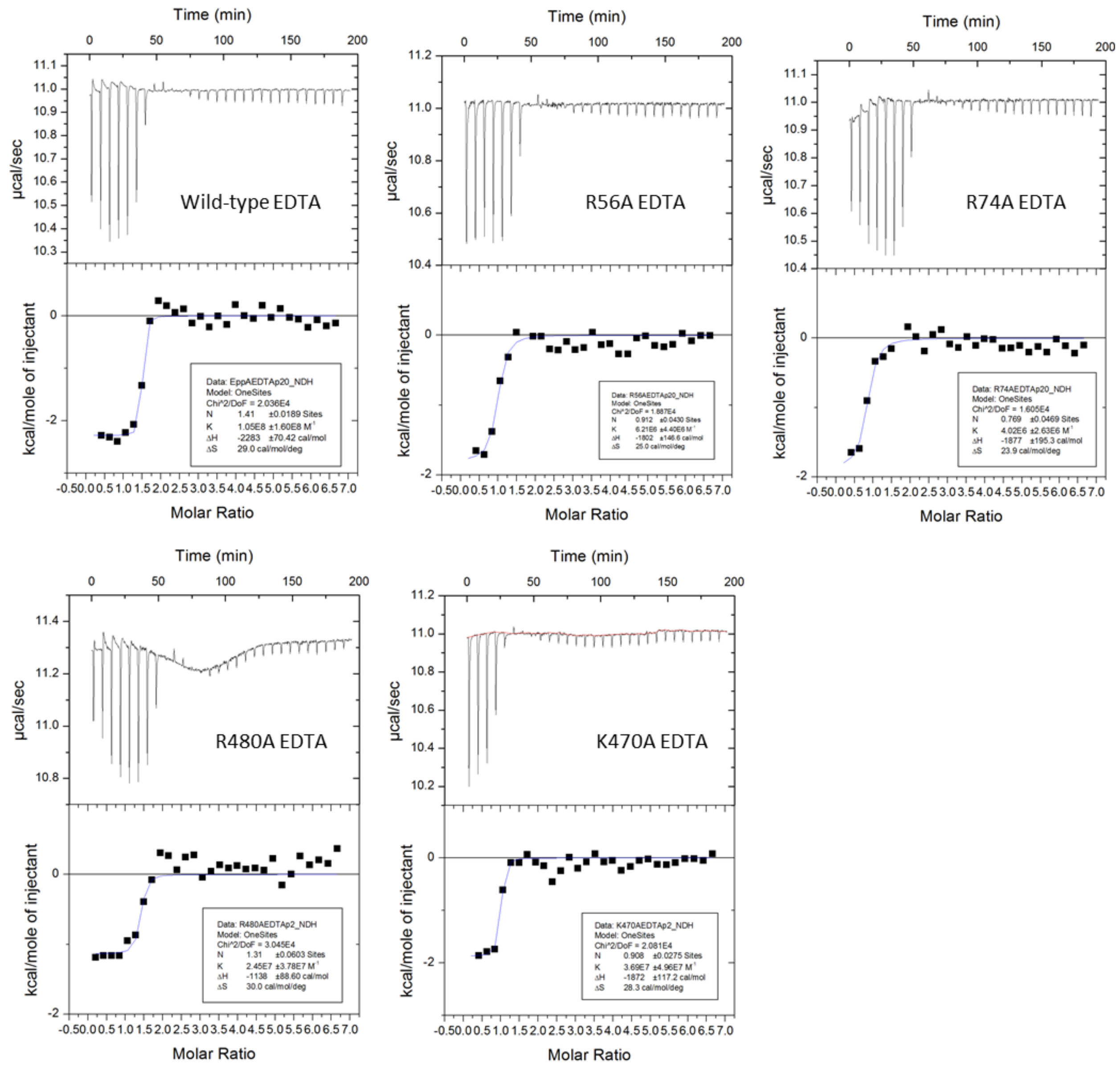

Figure S3: CLUSTAL Omega Multiple Sequence Alignment of Top Ten PSI-BLAST Matches

```
EppA  (6WM6) --------------------QDNLVTGEL------ITTVNSGTPGKGGEVTFAVTRD-IT 33
Lpqw  (2GRV) CTVSPPPAPQSTETTETTPPPPPKAPTQI------IMAIDSIGPGFN------------- 41
CBP   (2O7I) ----------------------------------QVSLPREDTVYIGGAL------WGPA 20
AgaA  (6HLX) ----------------------------------------------GGTLYFGLSGE-PS 13
OppA  (3DRF) -----------------------TSTKKLKAGNFDVAYQNPDKAIKGGNLKVAYQSDSPM 37
OppA2 (2WOK) ------------MTTAARRPAPTTAGAGWDA---GVGALVNPSRRRGGTLRLVSSAD-VD 44
MnBP3 (4PFT) -----------------------------------QTFERNKTLYWGGAL------WSPP 19
MoaA  (6TFX) ---------------------------------------------AKTELSMGVASEDVT 15
NikZ  (4OET) -------------------------------------------KIPKDTLIIAVENE-IA 16
CtaP  (5ISU) ---------------------------------SSDKANGSGKAKDGGSLIIGVTGD-PE 26
CoSBP (1ZTY) ---------------------------------------RSELTIVPDFY------PTMV 15


EppA  (6WM6) NWNVTSALGNN--AVVRTVTLPIVPSAFMVQPD------FTLKMNTDLLESAELTSTDPQ 85
Lpqw  (2GRV) ---PHLLSDQS--PVNAAIASLVLPSSFRPVPDPTSPTGSRWELDTTLLESAEVTNENPF 96
CBP   (2O7I) TTWNLYAPQST-WGTDQFMYLPAFQYDLGRDA----------W-IPVIAERYEFV-DDK- 66
AgaA  (6HLX) TLDTVVQPGTSGRTVKLAIHRGLVNYG----IDG--------KISPELAESYEVS-PDAK 60
OppA  (3DRF) K--AQWLSGLSNDAT----FATM--SGPGGGQDGLFFTDSGFKFIKGGAADVALD-KESK 88
OppA2 (2WOK) SLDPARTY-----YVWVWLLQRLLNRTLMAYPTDPGP--AGLVPAPDLAEGPGEVSDGGR 97
MnBP3 (4PFT) SNWNPFTPWNAVAGTIGLVYEPLFLYDPLNDK----------F-EPWLAEKGEWV-SNN- 66
MoaA  (6TFX) TLDPHFATTTSDRTLVSYIYGALVRFAPGSANPS--------SIEADLAESWESN-ADQL 66
NikZ  (4OET) RINPAYSEDHD--AVINLVFSGLTRFD----ENM--------SLKPDLAKSWDIS-KDGL 61
CtaP  (5ISU) VINPNYASDRV--TLTI-QQAVYAPLF----WEV--------DGKPALAKSLDIS-DDNL 70
CoSBP (1ZTY) RNFNPYLATNL-RTTTDFIYEPLVVFNEMKGN----------TPVFRLAESYKMA-DDLM 63


EppA  (6WM6) TVVYRIREDAVWSDGV-PITGDDFIYFWKTQNRRDCPECL--------------INASYG 130
Lpqw  (2GRV) TVTYKIRPEAQWTDNA-PIAADDYWYLWRQMVSQP----G--------------VVDPAG 137
CBP   (2O7I) TLRIYIRPEARWSDGV-PITADDFVYALELTKEL--------------------GIGPGG 105
AgaA  (6HLX) EFTFHLRQAKFHDGT--TVTSADVKASLERILDPK-------------------GKASFR 99
OppA  (3DRF) TATITLRKDLKWSDGS-EVTAKDYEFTYETIANPA-------------------YGSDRW 128
OppA2 (2WOK) TWTYRLRRGLRYDDGT-PITSDDVRHAVQRVFAQDVLPGGPTYLIPLLDDPERPYPGPYR 156
MnBP3 (4PFT) EYVLTLRKGLRWQDGV-PLTADDVVFTFEIAKKYT-------------------GISYSP 106
MoaA  (6TFX) VWTFKLRPDVKWQGGYGNVTADDVVFSLDKARDPK-------------------R-SAFS 106
NikZ  (4OET) VYDIFLRDDVLWHDGV-KFSADDVKFSIEAFKNPK-------------------NNSSIY 101
CtaP  (5ISU) TYTVKLKDGLTWHDGK-PLTADDVVFTVNSILDTK-------------------QNSPNR 110
CoSBP (1ZTY) SVTFDIRKGVKWSDGE-AFTADDVVYSFGLLKAKP-------------------ELDQR- 102
                  ::      .    .:. *

EppA  (6WM6) HDF--------------IETLEQDE-----TGKVVTATFSEPFLGWQGLF-----MFLYP 166
Lpqw  (2GRV) YDL--------------ITGVQSVE-----GGKQAVVTFSQPYPAWRELF-----NDILP 173
CBP   (2O7I) GW---------------DTYIEYVKA---VDTKVVEFKAKEENLNYFQFLSYSLGAQPMP 147
AgaA  (6HLX) NEL---------------SSISKIET---PDEKTVKLTLSTPSVAMIDYL-ALPESVIVP 140
OppA  (3DRF) TDSLANIVGLSDYHTGKAKTISGITFPDGENGKVIKVQFKEMKPGMTQSGNGYFLETVAP 188
OppA2 (2WOK) TD----------------EPLRSVLT---PDEHTIVFRLTRPFSDFDHLM-----AQPCA 192
MnBP3 (4PFT) VW---------------N-WLGRIER---VDERTLKFVFSDP--RYQEWKQMLINTPIVP 145
MoaA  (6TFX) GDY---------------AAIQKVEA---VDAKTVRITLTRRVPSLLALLSNFSGGFIIP 148
NikZ  (4OET) VNF---------------EDIKSVEI---LNPSHVKITLFKPYPAFLDALS----IGMLP 139
CtaP  (5ISU) GNFVFD------------DKPVKVEA---VDDTTVKFTLPTVAPAFENTIK---TFFPIP 152
CoSBP (1ZTY) GI---------------NKWVTSVEK---VDEYKVRFRLSEANSNVPYEI---SLIPIVA 141


EppA  (6WM6) AHLAEKHGDIAESYNNFLSNEVPAWSGGPYMVESFDPGQLVTLVPNP--KWYGEKGPYLD 224
Lpqw  (2GRV) AHIVKD---IPGGFGAGLARAMP-VTGGQFRVETIDPQRDEILLARNDRFWSVPAKPD-- 227
CBP   (2O7I) KHVYERIRAQMNIKDWINDKPEEQVVSGPYKL-YYYDPNIVVYQRVDDW-WGKDIFG-L- 203
AgaA  (6HLX) AAWLAK-------N--ADNPNAAPVGAGPFKFAGWTRGREITVKKFDDYYKKG------K 185
OppA  (3DRF) YQYLKDVAPKDLAS--SPKTTTKPLVTGPFKPENVVAGESIKYVPNPYY-WGE------K 239
OppA2 (2WOK) APVPR-------RSDTGADYGRDPRSSGPYRVARHEPDTLLHLERNP--HWDRATDPIRP 243
MnBP3 (4PFT) KHIWENKTEE----EVLQAANENPVGSGPYYV-ESWADDRCVFKKNGNW-WGIRELG-YD 198
```

```
MoaA  (6TFX) KKAFKE-------R--GDDFKRRPVGFGPFQVESIQPGQSVTLTANAEY-FRG------K 192
NikZ  (4OET) KHLLE-NE---NLN--TSSFNQNPIGTGPYKFVKWKKGEYVEFKANEHF-YLD------K 186
CtaP  (5ISU) KHIFEGVE---NIE--KSDKNKNPIGSGPYKFVEYKTGEYVSLERFNDY-FDG------K 200
CoSBP (1ZTY) EHVWKDVKDP------TTFTNENPVGTGPFTVIDTFTPQLYIQCRNPNY-WDAANLE-VD 193
                                        * :

EppA  (6WM6) ----KLKFRIITDSTQQLTALENGEVDVIYPQGATQDM---VEQAAGLDYLGIDFQMNPS 277
Lpqw  (2GRV) ----LVLFRRGGAPAALADSIRNGDTQVAQVHGGAATF---AQLSAIPDV-RTARIVTP- 278
CBP   (2O7I) PRPKYLAHVIYKDNPSASLAFERGDIDWNGLFIPSVWE---LWEKKGLPVGTW-YKKEPY 259
AgaA  (6HLX) PDLDEVHYVFYSDENTRVNALKSGDVDIIDYVPAKDAA-DIAKGPET-QLL-R--NTGPF 240
OppA  (3DRF) PKLNSITYEVVSTA-KSVAALSSSKYDIINGMVSSQYK--QVKNLKGYKVLGQ--QAMYI 294
OppA2 (2WOK) ALPDRVELTIGLDVDVLDARLIAGEFDINLEGRGLQHA---AQRRATADEVLRSHTDNPR 300
MnBP3 (4PFT) PKPERIVELRVLSNNVAVGMLMKGELDWSNFFLPGVPV---LK--KAYGIVTW-YENAPY 252
MoaA  (6TFX) PKLSKISYRFLNNEAARDLAFESGELDVEQGNQDQRWLQRLTANPEN-VVDTI--EPAEL 249
NikZ  (4OET) VKTPRLIIKHIFDPSIASAELKNGKIDAALIDVSLLN---IFKNDENFGILRE--KSADY 241
CtaP  (5ISU) PKLDKVTFRITKDQNAANLALQNGEINLKSIQPSDRN---KVEKASAVNIITY--PENRL 255
CoSBP (1ZTY) C----LRVPQIANNDQLLGKIVNSELDWTSSFVPDIDR---TYAAANPNHHYW-YPAA-- 243
                  :              :  .. :

EppA  (6WM6) ---------A-NWYFMGLNS------KAGPMSDIALRKAVLTAIDAGDLKAKTADPYLRN 321
Lpqw  (2GRV) -----------RVMQLTLRA------QQPKLADPQVRKAILGLIDVDLLASVGAGDD-NT 320
CBP   (2O7I) F-------IPDGVGFVYVNN------TKPGLSDPAVRKAIAYAIPYNEMLKKAYFGYG-- 304
AgaA  (6HLX) -------------MGLQFNT------KFEPFSKPEVRQAIAYAVDRSAIINTAFNGLG-- 279
OppA  (3DRF) SLMYYNLGHYDAKNSINVQD------RKTPLQDQNVRQAIGYARNVAEVDNKFSNGLS-- 346
OppA2 (2WOK) ---------TSFLHFVAMQP------HIPPFDNVHVRRAVQYAADKILLQDARGGPVN-G 344
MnBP3 (4PFT) M-------LPANTAGIYINV------NKYPLSIPEFRRAMAYAINPEKIVTRAYENMV-- 297
MoaA  (6TFX) -------------NLLHINI------TKPPFNDIRVRQALAHTVNAAQIAKYRGERVN-- 288
NikZ  (4OET) -------------RALMFNL------DNEFLKDLKVRQALNYAVDKESIVKNLLHDYA-- 280
CtaP  (5ISU) -------------SYATFNE------NQPALKSKELRQALSYALDREEIIDAAYGSDE-- 294
CoSBP (1ZTY) -----------GTQAFMVNFKNPDPAKKEALDNVDFRRAFSMALDRQTIIDIAFYGSG-- 290
                              ..           :    .*:*.        :

EppA  (6WM6) WPHMGSVMFLPNQAGYA-----------DRRGARGYGTGDVEKAKGILSEAGYKLSGGS- 369
Lpqw  (2GRV) VTLAQAQVRSPSDPGYV-----------PTAPPA----MTRDDALELLRDAGYVSEPVPP 365
CBP   (2O7I) -SQAHPSM-VIDLFEPYKQYIDYELAKKTFGTEDGRIPFDLDMANKILDEAGYKKG---- 358
AgaA  (6HLX) -SPIFGIA-IPKG----YM--------GYSDAKANYFSHHVEKAKALLAKAGYPNGF--- 322
OppA  (3DRF) -TPANSLI-PPIF----KQ--------FTSSSVKGYEKQDLDKANKLLDEDGWKLNK--- 389
OppA2 (2WOK) G-DLTTALFPPTLPAHQD----LDL--YPTGP-D--LRGDLDAARAELAAAGL------- 387
MnBP3 (4PFT) -TAANPAG-ILPLPGYMKYY-PKEV------VDKYGFKYDPEMAKKILDELGFKDVN--- 345
MoaA  (6TFX) --RAVPSV-IPSN----NL--------GFDP-DAGVLNYDPAQSKKLLAEAGFPNGV--- 329
NikZ  (4OET) -FVANH---PLE-----RS--------WANSKNFKIYKYDPKKAEDLLVSAGFKKNK--- 320
CtaP  (5ISU) -YAKPASS-FLTE----NT--------KYFTDKVETYDQDIAKAKKLVKESGFDTS---- 336
CoSBP (1ZTY) -TVNDFAS-GLGY--AFEAWSDE----ATHKKYKGFNTYDVEGSKKLLAKAGFKDVN--- 339
                                                        :   :   *

EppA  (6WM6) ---------------LLDPSGKPVSTLRLSFPPGYPAANDMARLITGYIAPLGLKTDLLT 414
Lpqw  (2GRV) PDNTADDPPPDNGRERIVKDGVPLT-IVLGVASNDPTSVAVANTAADQLRNVGIDASVLA 424
CBP   (2O7I) -----------PDGVRVGPDGTKLGPYTISVPYGWTDWMMMCEMIAKNLRSIGIDVKTEF 407
AgaA  (6HLX) -------------------------EVRLLATSQYSFQQNTAIAIQSELAKIGIKVKLD- 356
OppA  (3DRF) -----------STGYREK-DGKEL-SLVYAARVGDANAETIAQNYIQQWKKIGVKVSLYN 436
OppA2 (2WOK) PDGFRAVIGTQRGKFR----------------------LVADAVVESLARVGIELTVKE 424
MnBP3 (4PFT) -----------KDGFREDPNGKPF-KLTIECPYGWTDWMVSIQSIAEDLVKVGINVEPKY 393
MoaA  (6TFX) -------------------------TVTMVASQLPGLES-LAQLIQAQVAEGGFTLNLQP 363
NikZ  (4OET) -----------DGNF-EK-DGKIL-EFEIWAMSNDPLRVSLAGILQSEFRKIGVVSKVVA 366
CtaP  (5ISU) --------------------Q----KLTVYYLNNSKSQESIALYLQQQYKEIGVTLDLKP 372
CoSBP (1ZTY) -----------GDGFVETPSGKSF-ELLIQSPNGWTDFNNTVQLAVEQLQEVGIKAKART 387
                                                            *.

EppA  (6WM6) G-PN---ATADYLLSG----NFDLHLNYFS----------------------Q------ 437
Lpqw  (2GRV) LDPV--ALYGDALVNN----RVDAVVGWRQAGG-DL-ATV-------LASRYGCRALEAT 469
CBP   (2O7I) P---DFSVWADRMTKG----TFDLIISWSVGPSFDHPFN-------------------- 439
```

```
AgaA  (6HLX) --L---PDWASRMAKA-STGDYDFTVMGSLGEI--TDAD--------------------- 387
OppA  (3DRF) GKLMEFNSWVDHMTTPPGANDWDITDGS-------------------------------- 464
OppA2 (2WOK) LDVA----------------------TYFSLGA-GHPETVREHGLGLLVTDWGAD----- 456
MnBP3 (4PFT) P---DYSKYADDLYGG----KFDLILNNFTTGVSATIWS--------------------- 425
MoaA  (6TFX) VEH---AAWHQMIRKD-LS---PIVLYGA-ARF--PIAD--------------------- 392
NikZ  (4OET) KPA---GSFDYSKVDS--------FLIGWG-SP--LDPD--------------------- 391
CtaP  (5ISU) TDP---NALSNITLDR-KNADYSIALNGYI-MG--NDPD--------------------- 404
CoSBP (1ZTY) P---EFAVYNQAMLEG----TYDVAYTNYFHGA--DPFT--------------------- 417


EppA  (6WM6) -----------------------------------QVFPAVKAGQIFLRDTRQNYFGFND 462
Lpqw  (2GRV) PVATAVPGPATTTSQAPTTTTTTTPPATTTPTP-TAPIPAPESGELV--QAPSNITGICD 526
CBP   (2O7I) --------------------------------IYRFVLDKRLSKPVGEVTWAGDWERYDN 467
AgaA  (6HLX) ---------------------------------WLSNYYYGGD-KLVRTNNSPYFND--Q 411
OppA  (3DRF) ---------------------------------WSLASEPSQQ-DLFSAAAPYNFGHFND 490
OppA2 (2WOK) -----------------------------FPTEYGFLAPLVDGRQIKRNGGNWNLPELDD 487
MnBP3 (4PFT) --------------------------------YFNGVFY--PDAVESEYSYSGNFGKYAN 451
MoaA  (6TFX) --------------------------------YYLTQFYHSAS-EIGKPTQVVNFSH--C 417
NikZ  (4OET) --------------------------------FHTFRVFESSQ-DSALNDEGWNFGHYHD 418
CtaP  (5ISU) ----------------------------------------AYK-SLYLSDAPYNYSNYHN 423
CoSBP (1ZTY) --------------------------------YWNSGYNSALQSGDGMPRFA--MHYFTD 443


EppA  (6WM6) PKIEEIIGKAA--AASSIEESAAILSEADELAMDYAALFPIYQLPTALIYKEAI-LNLR- 518
Lpqw  (2GRV) RSIQPRIDAAL----DGTDDIADVIQAVEPRLWNMATVLPILQDTTIVAAGPSV-QNVSL 581
CBP   (2O7I) DEVVELLDKAV--STLDPEVRKQAYFRIQQIIYRDMPSIPAFYTAHWYEYSTKYWINWPS 525
AgaA  (6HLX) QIN-DLLDKGR--ATVDKAERVKIYDAFVDRALELSPLVYFMWREQNYAVKKGV-TGFTN 467
OppA  (3DRF) SEITKDLNDIDSAKSENPTYRKAAFVKYQEDMNKKAYVIPTNFMLNYTPVNKRV-VGMTL 549
OppA2 (2WOK) PEVNALIDETL--HTTDPAARAELWRAVERRVMEHAVLLPLVHDKTLHFRNPWV-TNVYV 544
MnBP3 (4PFT) PEVETLLDELN--RSNDDAKIKEVVAKLSEILLKDLPFIPLWYNGAWFQASEAVWTNWPT 509
MoaA  (6TFX) NVADKQIEAAR--TETDPNKQIE------------------LWKEAQKLIVSNV-CAIPL 456
NikZ  (4OET) KKVDIALQKAR--NTSNLEERKKYYKDFIDALYENPPFIFLAYLDFALVYNKDL-KGIKT 475
CtaP  (5ISU) KDLDALWEKGA--VTADDKERQEIYEKIQNTIADDAVIYPISYDNAVLALDSRY-GGQKA 480
CoSBP (1ZTY) KKLDGLLDSFY--KTADKNEQLAIAHGIQKIIAENQVTIPVMSGAWMYQYNTTRFTGWWS 501
                             .

EppA  (6WM6) DNPNQLGP---AY----NTAEWG----LAE------------------------------ 537
Lpqw  (2GRV) TGAVPVGI---VG----DAGDWT----KTK------------------------------ 600
CBP   (2O7I) ED-NPAWFRPSPW----HADAWPTLFIISKKSDPQPVPSWLGTVDEGGIEIPTAKIFEDL 580
AgaA  (6HLX) ---MP-GF-L-TF-------QSG----LSIEN----------------------TKIE-- 486
OppA  (3DRF) D---Y-GA-MNTWSEI----GVS-------------SAKL----------ATK------- 570
OppA2 (2WOK) HPAFG------LY----DIQAMG----LAEE-----------------------D----- 562
MnBP3 (4PFT) EK-NPYAV-PIGWNGWWQLTGIKTLFGIEAK----------------------------- 538
MoaA  (6TFX) ---TE-NL-G-TWARKNKL-GWG----FELKGS-MPSAPL----------ITEQTYFKD- 493
NikZ  (4OET) RTLGHHGV-GFTWNVY----EWS--------------K---------------------- 494
CtaP  (5ISU) ATPQP--V-TMF---R----DLS--------------KLY----------LTE------- 499
CoSBP (1ZTY) EE-NPKGR-PSVWAGIPER----LLHVLDLK----PVK--------------------- 529


EppA  (6WM6) -----
Lpqw  (2GRV) -----
CBP   (2O7I) QKATM 585
AgaA  (6HLX) -----
OppA  (3DRF) -----
OppA2 (2WOK) -----
MnBP3 (4PFT) -----
MoaA  (6TFX) -----
NikZ  (4OET) -----
CtaP  (5ISU) -----
CoSBP (1ZTY) -----
```

Figure S4: CLUSTAL Omega Multiple Sequence Alignment of Top Ten DALI Results

```
EppA (6WM6) -----------------QDNLV------TGELITTVNSGTPGKGGEVTFAVTRDITNWNV 37
NIKZ (4OET) -----------------------------------------IPKDTLIIAVENEIARINP 19
APPA (1XOC) -----------------------CSGSKSSNSSAKKSAGKPQQGGDLVVGSIGEPTLFNS 37
CTAP (5ISU) ------------------------------SSDKANGSGKAKDGGSLIIGVTGDPEVINP 30
PBP  (5U4O) MRFKRGLVSCFIAILCLSVFLAGCSSNAKTG-NEGSGSKGTKEGGVLTIARLSDADNLDP 59
YLIB (1UQW) -------------------------------------------AKDVVVAVGSNFTTLDP 17
GBPA (3M8U) ------------------------------------------ADKTFINCVSRSPTGFSP 18
DPPA (4QFO) ------MHKLLL--ALLSLSLVGCI-DSKEE---IL----EEKNQGLVYCAEANPVSFNP 44
PBP  (3RQT) -----------------------CGSM------------HSSGKDLNISLPLKTKSIAP 24
DPPA (1DPP) --------------------------------------------KTLVYCSEGSPEGFNP 16
DPPA (6PU3) ------------------ENPNATLNPSKEN---ISVKEQKRFGGVLVFARGADGSSMDP 39
                                                          .      .

EppA (6WM6) TSALGNNAVVRTVTLPIVPSAFMVQPDF-TLKMNTDLLESAELTSTDPQTVVYRIREDAV 96
NIKZ (4OET) AYSEDHD----A-VINL-VFSGLTRFDE-NMSLKPDLAKSWD-ISKDGLVYDIFLRDDVL 71
APPA (1XOC) LYSTDDAST--D-IENM-LYSFLTKTDE-KLNVKLSLAESIK-ELDGGLAYDVKIKKGVK 91
CTAP (5ISU) NYASDRVTL--T-IQQA-VYAPLFW----EVDGKPALAKSLD-ISDDNLTYTVKLKDGLT 81
PBP  (5U4O) HFITNIPSA--SVVYHK-VYENLVQRDK-NMDFKPMLAKEWK-QI-DDLNWEFKLQQGVT 113
YLIB (1UQW) YDANDTLS---QAVAKS-FYQGLFGLDK-EMKLKNVLAESYT-VSDDGITYTVKLREGIK 71
GBPA (3M8U) ALVMDGISY--NASSQQ-VYNRLVEFKRGSTDIEPALAESWT-VSDDGLTYTFNLRKGVK 74
DPPA (4QFO) QVTTTGSTI--DIIANQ-LYDRLISIDPVTAEFKSELATDWK-ISKDGKSVTFTLRKGVK 100
PBP  (3RQT) YETDVPVKI--G------AAESLFKTND-QGKIEKALVKSYH-QP-NDTTLDIELKDNIK 73
DPPA (1DPP) QLFTSGTTY--DASSVP-LYNRLVEFKIGTTEVIPGLAEKWE-VSEDGKTYTFHLRKGVK 72
DPPA (6PU3) ALVTDGESY--VAT-GN-IYDTLVQFKYGTTEIEPALATSWD-ISPDGLVYTFHLRKGVY 94
                                  :        .    *  .      .       ::..

EppA (6WM6) WSDGV------PITGDDFIYFWKTQNRRDCPECLI-NASY------G-HDFIETLEQ--D 140
NIKZ (4OET) WHDGV------KFSADDVKFSIEAFKNPKNNSS-----IY------VNFEDIKSVEILNP 114
APPA (1XOC) FHDGK------ELTADDVVFTYSVPLSKDYKGER--GSTY------EML---KSVEKKGD 134
CTAP (5ISU) WHDGK------PLTADDVVFTVNSILDTKQNSPN--RGNF------VFDDKPVKVEAVDD 127
PBP  (5U4O) FQDGA------PFNAEAVKKNFERVLDPKVGSNR-----------ATVYSMIQEIKVIDE 156
YLIB (1UQW) FQDGT------DFNAAAVKANLDRASDPANHLKR-----------YNLYKNIAKTEAIDP 114
GBPA (3M8U) FHSNKEFTPSRDFNADDVVFSFQRQLDPNHPYHNVSKATYPYFKAMKFPTLLKSVEKVDD 134
DPPA (4QFO) FHTTAYFTPTREFNADDVIFTFSRLFDVYNPYHFVGDANYPYFQSVGIDQLIRKIVRVSD 160
PBP  (3RQT) FQNGQ------KLTAEKVKSSLENSMKKSDLVKY-------------SLPISSITA-KG 112
DPPA (1DPP) WHDNKEFKPTRELNADDVVFSFDRQKNAQNPYHKVSGGSYEYFEGMGLPELISEVKKVDD 132
DPPA (6PU3) FHQTKYWNKKVEFSAKDVLFSFERQMDKAKRYYSPGAKSYKYWEGMGMSHIIKSIEALDD 154
            :           :..  .    .

EppA (6WM6) ETGKVVTATFSEPFLGWQ-GLFMFLYPAHLAEKHGDIAESYNNFLSNEVPAWSGGPYMVE 199
NIKZ (4OET) SHVKITLFKPY-PAFLDA-L-SIGMLPKHLLENENLN----TSS--FNQNPIGTGPYKFV 165
APPA (1XOC) YEVLFKLKYKDGNFYNNA-LDSTAILPKHILGNVPIADLEENEF--NRKKPIGSGPFKFK 191
CTAP (5ISU) TTVKFTLPTVA-PAFENT-IKTFFPIPKHIFEGVENI---EKSD--KNKNPIGSGPYKFV 180
PBP  (5U4O) YTVQFILKYPYSPLLSIFASNEGSILSPKAID-------EKGKG--LAQHPVGTGPYTFK 207
YLIB (1UQW) TTVKITLKQPFSAFINILAHPATAMISPAALE-------KYGKE--IGFYPVGTGPYELD 165
GBPA (3M8U) HTVKITLNRQDATFLASLGMDFISIYSAEYADKMLAA--GKPET--IDTTPIGTGPFVFA 190
DPPA (4QFO) HQVRFELFNAESSFLANMATDFAVVLSKEYAMALKAN--NQENL--FDQYPVGTGPYIYK 216
PBP  (3RQT) QKLTIKTNSAYPELVSELANPFMAIYDT-----------DAKSD--VNQTPVGTGPYQIK 159
DPPA (1DPP) NTVQFVLTRPEAPFLADLAMDFASILSKEYADAMMKA--GTPEK--LDLNPIGTGPFQLQ 188
DPPA (6PU3) YTIRFTLNGPEAPFLANLGMDFLSILSKDYADYLEQN--NKKDE--LAKKPVGTGPFKFF 210
                .                                     .         . **:

EppA (6WM6) SFDPGQLVTLVPNPKWYGEKGPYLDKLKFRIITDSTQQLTALENGEVDVIY-PQGATQDM 258
NIKZ (4OET) KWKKGEYVEFKANEHFYLDK-VKTPRLIIKHIFDPSIASAELKNGKIDAAL-IDVSLLNI 223
```

```
APPA (1XOC) EWKQGQYIKLEANDDYFEGR-PYLDTVTYKVIPDANAAVAQLQAGDINFFN-VPATDYKT 249
CTAP (5ISU) EYKTGEYVSLERFNDYFDGK-PKLDKVTFRITKDQNAANLALQNGEINLKS-IQPSDRNK 238
PBP  (5U4O) SWKPGEEIRLEKNKNYWGEK-AKVDEVVFKVVPEDATRIGMIETSEAHIAENLPVTEVER 266
YLIB (1UQW) TWNQTDFVKVKKFAGYWQPGLPKLDSITWRPVADNNTRAAMLQTGEAQFAFPIPYEQATL 225
GBPA (3M8U) GYQVDQKSRYFAHKEYWKGK-ADIDRLIFEIVPDATARYAKLQAGACDLIDFPNAADLEK 249
DPPA (4QFO) EYRRDHLVRFYKNADYWKHE-VALEQLVYDITPNGTTRIAKILTKECDVTAHPSSAQLSI 275
PBP  (3RQT) DYKQSRKISLSNFKDYWQGK-PKLDHITVTYQEDGNNRVRNLESQKDDLITDVPVNKVQD 218
DPPA (1DPP) QYQKDSRIRYKAFDGYWGTK-PQIDTLVFSITPDASVRYAKLQKNECQVMPYPNPADIAR 247
DPPA (6PU3) LWNKDEKIILLKNQDYWGPK-AYLDKVVVRTIPNSSTRALALRTGEIMLMTGPNLNEVEQ 269
             :             ::         :      :       :

EppA (6WM6) VEQAAGLDYLGIDFQMNPSANWYFMGLNSKAGPMSDIALRKAVLTAIDAGDLKAKTADPY 318
NIKZ (4OET) FKNDEN-----FGILREKSADYRALMFNLDNEFLKDLKVRQALNYAVDKESIVKNLLHDY 278
APPA (1XOC) AEKFNN-----LKIVTDLALSYVYIGWNEKNELFKDKKVRQALTTALDRESIVSQVLDGD 304
CTAP (5ISU) VEKASA-----VNIITYPENRLSYATFNENQPALKSKELRQALSYALDREEIIDAAYGSD 293
PBP  (5U4O) VKNSPS-----MELIENEGLGVEYIGFNVEKKPFDNPLVRQAIAHAIETKGILKGVYNNV 321
YLIB (1UQW) LEKNKN-----IELMASPSIMQRYISMNVTQKPFDNPKVREALNYAINRPALVKVAFAGY 280
GBPA (3M8U) MKTDPK-----VNLLSQSGLNIAYIAFNTEKAPFDNVKVRQALNYAVDKNAIIDAVYRGA 304
DPPA (4QFO) LAQRDD-----INVERETNLNIGYWAFNTERPPFDNLKVRQALVHAIDIEKIMQAVYYGN 330
PBP  (3RQT) IENNQN-----LKVSKESGFRTSLLMYNHTNKKMT-KSVREALDHIIDRQGIADHIYQGY 272
DPPA (1DPP) MKQDKS-----INLMEMPGLNVGYLSYNVQKKPLDDVKVRQALTYAVNKDAIIKAVYQGA 302
DPPA (6PU3) LEKLPN-----IVVDKSAGLLASWLSLNTQKKYFNNPLVRLAINHAINVDDYIKVIYEGF 324
                       . .             *     :    :* *:   ::

EppA (6WM6) LRNWPHMGSVMFLPNQAGYAD--RRGARGYGTGDVEKAKGILSEAGYKLS-GGSLLDPSG 375
NIKZ (4OET) ----AFVANHPLE-RSWANSK----NFKI-YKYDPKKAEDLLVSAGFKK-NKDGNFEKDG 327
APPA (1XOC) ----GEVAYIPESPLSWNYPK--DIDVPK-FEYNEKKAKQMLAEAGWKDTNGDGILDKDG 357
CTAP (5ISU) ----EYAK--PASSFLTENTKYFTDKVET-YDQDIAKAKKLVKESGFDTSQKL------- 339
PBP  (5U4O) ----GTEINSVMTPKVFGYTK----DVKG-YKYDINTAKKLLADAGYPNG---------- 362
YLIB (1UQW) ----ATPATGVVPPSI-AYAQ----SYKP-WPYDPVKARELLKEAGYPNG---------- 320
GBPA (3M8U) ----GVAAKNPLPPTIWGYNN----EITG-YEYNPEKAKQLLKEAGFENG---------- 345
DPPA (4QFO) ----GLRARSILPPTSWAFEP----QKNM-PIFDPQLAKKLLTEAGYEKG---------- 371
PBP  (3RQT) ----AKPATSPFNDKI-PYIK----EPKL-TKQNIEQAKMLLAKDGYTKEHP-------- 314
DPPA (1DPP) ----GVSAKNLIPPTMWGYND----DVQD-YTYDPEKAKALLKEAGLEKG---------- 343
DPPA (6PU3) ----AQKMVNPFPPTIWGYNY----NIKP-YEYDLKKAKELLKQAGYPNG---------- 365
                                             :   *. :: . *

EppA (6WM6) KPVSTLRLSFP---PGYPAANDMARLITGYIAPLGLKTDLLTGPNATADYLL-------- 424
NIKZ (4OET) KILEFEIWAMSNDP----LRVSLAGILQSEFRKIGVVSKVVAKPA--GSFD--------- 372
APPA (1XOC) KKFSFTLKTNQGNK----VREDIAVVVQEQLKKIGIEVKTQIVEW--SALVE-Q--MNPP 408
CTAP (5ISU) -----TVYYLNNSK----SQESIALYLQQQYKEIGVTLDLKPTDP--NALSNIT--LDRK 386
PBP  (5U4O) --FKTTIWTNDSKV-----RMALVEVIQSQLKGIGVDVEIKVMEY--GAFLAA-----TN 408
YLIB (1UQW) --FSTTLWSSHNHS----TAQKVLQFTQQQLAQVGIKAQVTAMDA--GQRAAEVEGKGQK 372
GBPA (3M8U) --FETDIWVQPVVRASNPNPRRMAELVQSDWEKVGVKSKLVSYEW--GDYIKR-----TK 396
DPPA (4QFO) --FDMSIWAMPVSRIYNPNARKMAELMQSDLRKIGVNVNIVEYEW--NTFIQR-----IG 422
PBP  (3RQT) --LKIKLITYDGRP----ELSKIAQVLQSDAKKANIEIDIKSVDDI-EGYLKD-----RS 362
DPPA (1DPP) --FSIDLWAMPVQRPYNPNARRMAEMIQADWAKVGVQAKIVTYEW--GEYLKR-----AK 394
DPPA (6PU3) --FKTTIFTTSTRN------PKGAVFIQASLAKIGIDVKIEVYEW--GAYLKR-----TG 410
                                              .:  .

EppA (6WM6) SGNFDLHL----------NYFSQQVFPAVKAGQIFLRDTRQNYFGFNDPKIEEIIGKAAA 474
NIKZ (4OET) YSKVDSFLIGWGSPL-DPDFHTFRVFESSQD--SALNDEGWNFGHYHDKKVDIALQKARN 429
APPA (1XOC) NWDFDAMVMGWSLST-FPDQ--YDIFHSSQ------IKKGLNYVWYKNAEADKLMKDAKS 459
CTAP (5ISU) NADYSIALNGYIMGN-DPDA--YKSLYLSD-------APYNYSNYHNKDLDALWEKGAV 435
PBP  (5U4O) KSEHAMFVGGWGNATGDGDYNQYNLFHSSSH--G----ATGNQFFYSNPEVDKLIEEARK 462
YLIB (1UQW) ESGVRMFYTGWSASTGEADWALSPLFASQNW--P---PTLFNTAFYSNKQVDDFLAQALK 427
GBPA (3M8U) AGELTAGTYGWSGDNGDPDNFLSPLFGSENV-------GNSNYARFKNPELDALLHKAVG 449
```

```
DPPA (4QFO) EHRHDSVLLGWAADTPDPDNFFSPLLSCTAT--F----SGKNPANWCNPEFDLLLTKALD 476
PBP  (3RQT) AWDATMYSFGTI-PRGDTGYFFNQAYKKD---------GAINKGDYNNSNVDDLINQLNH 412
DPPA (1DPP) DGEHQTVMMGWTGDNGDPDNFFATLFSCAAS--E----QGSNYSKWCYKPFEDLIQPARA 448
DPPA (6PU3) LGEHEMAFAGWMADIADPDNFLYTLWSKQAA--S--AIPTQNGSFYKSDAFSDLLIKAKR 466
                              .                      *   :     .

EppA (6WM6) ASSIEESAAILSEADELAMDYAALFPIYQLPTALIYKEAILNLRDN---PNQLGPAYNTA 531
NIKZ (4OET) TSNLEERKKYYKDFIDALYENPPFIFLAYLDFALVYNKDLKGIKTRTLGHHGVGFTWNVY 489
APPA (1XOC) ISDRKQYSKEYEQIYQKIAEDQPYTFLYYPNNHMAMPENLEGYKYH---PK--RDLYNIE 514
CTAP (5ISU) TADDKERQEIYEKIQNTIADDAVIYPISYDNAVLALDSRYGGQKAATPQPV--TMFRDLS 493
PBP  (5U4O) EKDETKRKELYKQLQEIELKDALLVPIRGINHIAATTKNIKGFWID---PSGYLRLEGVE 519
YLIB (1UQW) TNDPAEKTRLYKAAQDIIWQESPWIPLVVEKLVSAHSKNLTGFWIM---PDTGFSFEDAD 484
GBPA (3M8U) LSDKAERAKIYEQAQVLLKEQAPWINVAHSINFAPTSKRVQDYKQS---PFGYTYLYGTK 506
DPPA (4QFO) TTDLNLRKQYYDAAQSMIIEQLPLYPIAHGMRFQASSADVEGITLG---PFGAISLANAR 533
PBP  (3RQT) TVDVKERHNISNDIIKLSSRDVPNSYIAYNDQIVAANSKVKNYKVT---PEGIYLIDYRT 469
DPPA (1DPP) TDDHNKRVELYKQAQVVMHDQAPALIIAHSTVFEPVRKEVKGYVVD---PLGKHHFENVS 505
DPPA (6PU3) VSDQKEREALYLKAQEIIHKDAPYVPLAYPYSVVPHLSKVKGYKTT---GVSVNRFFKVY 523
              .                       :              .

EppA (6WM6) EWGLAE 537
NIKZ (4OET) EWSK-- 493
APPA (1XOC) KWWLAK 520
CTAP (5ISU) KLYLTE 499
PBP  (5U4O) LQ---- 521
YLIB (1UQW) LQ---- 486
GBPA (3M8U) LAD--- 509
DPPA (4QFO) KK---- 535
PBP  (3RQT) TIER-- 473
DPPA (1DPP) IE---- 507
DPPA (6PU3) LEK--- 526
```

Figure S5. Docking of MgEDTA to EppA.

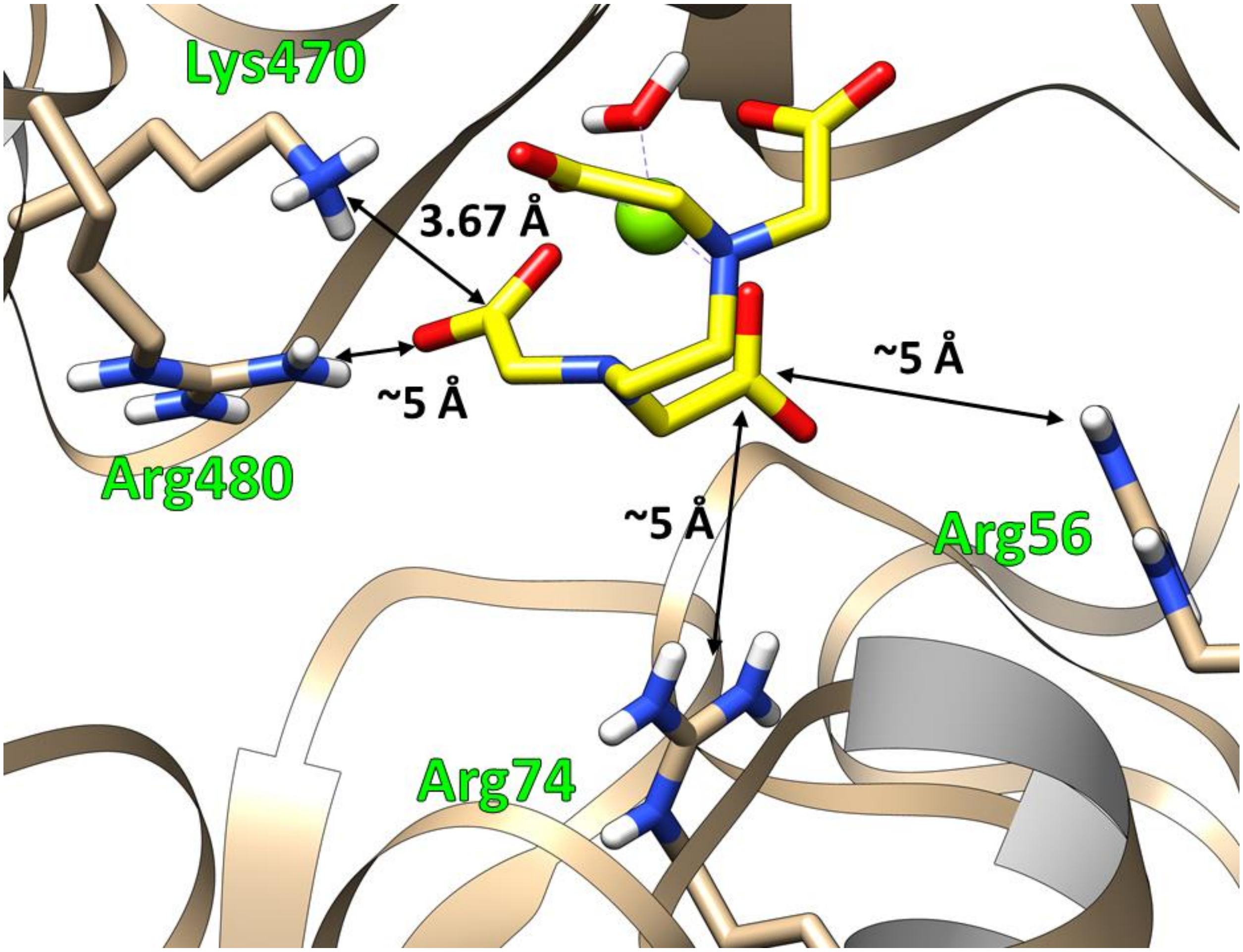

Table S1. Structural Homology by Pairwise Secondary Structure Superposition of Top Ten PSI-BLAST Results with EppA.

Name	PDB	Source	rmsd A (Å)	rmsd B (Å)	rmsd C (Å)
LpqW	2GRV	*Mycobacterium smegmatis*	4.3784	4.3395	4.3845
CBP	2O7I	*Thermotoga maritima*	3.1868	n/a	n/a
AgaA	6HLX	*Rhizobium radiobacter*	3.152	n/a	n/a
OppA	3DRF	*Lactococcus lactis*	3.0335	n/a	n/a
OppA2	2WOK	*Streptomyces clavuligerus*	2.7775	n/a	n/a
MnBP3	4PFT	*Thermotoga maritima*	2.8546	2.9576	n/a
MoaA	6TFX	*Rhizobium radiobacter*	2.751	2.7528	n/a
NikZ	4OET	*Campylobacter jejuni*	2.3367	2.254	2.29535
CtaP	5ISU	*Listeria monocytogenes*	2.3286	n/a	n/a
CosBP	1ZTY	*Vibrio cholerae*	3.8456	n/a	n/a

Table S2. Structural Homology by Pairwise Secondary Structure Superposition of Top Ten DALI Results with EppA.

Name	PDB	Source	rmsd A (Å)	rmsd B (Å)	rmsd C (Å)	rmsd D (Å)
NikZ	4OET	*Campylobacter jejuni*	2.3367	2.254	n/a	n/a
AppA	1XOC	*Bacillus subtilis*	2.8577	n/a	n/a	n/a
CtaP	5ISU	*Listeria monocytogenes*	n/a	n/a	n/a	n/a
PBP	5U4O	*Bacillus anthracis* str. Ames	2.6672	n/a	n/a	n/a
YliB	1UQW	*Escherichia coli*	2.9161	2.8733	n/a	n/a
GbpA	3M8U	*Haemophilus parasuis*	2.7542	n/a	n/a	n/a
DppA	4QFO	*Pseudoalteromonas* sp. SM9913	2.6678	2.6334	n/a	n/a
PBP	3RQT	*Staphylococcus aureus*	2.976	n/a	n/a	n/a
DppA	1DPP	*Escherichia coli*	3.1429	3.1429	3.1429	3.1428
DppA	6PU3	*Helicobacter pylori*	2.7977	n/a	n/a	n/a

Table S3. ConSurf amino acid conservation scores

POS	SEQ	RESID	SCORE	COLOR	CONF. INT.	CS	MSA	RESIDUE VARIETY
31	V	VAL	-0.124	5*	-0.857,0.318	8,4	4/30	V,I
32	T	THR	-0.648	7*	-1.269,-0.258	9,6	3/30	T
33	G	GLY	-0.074	5	-0.727,0.318	7,4	8/30	T,G,A
34	E	GLU	2.38	1	1.140,3.442	1,1	12/30	T,E,S,G,P,Q
35	L	LEU	0.697	3*	-0.058,1.140	5,1	13/30	D,T,N,L
36	I	ILE	0.405	4*	-0.347,0.872	6,2	13/30	L,P,I,A
37	T	THR	-1.11	9	-1.388,-0.979	9,8	28/30	N,K,T
38	T	THR	-0.036	5	-0.511,0.318	7,4	28/30	D,S,A,E,T,N
39	V	VAL	-0.975	8	-1.327,-0.793	9,8	28/30	V,C
40	N	ASN	-1.403	9	-1.589,-1.327	9,9	30/30	N
41	S	SER	0.245	4	-0.258,0.656	6,3	30/30	T,E,A,S,G
42	G	GLY	0.304	4	-0.258,0.656	6,3	30/30	V,A,S,G
43	T	THR	-0.573	7	-0.919,-0.347	8,6	30/30	T,E,K,Q
44	P	PRO	1.236	1	0.318,2.041	4,1	30/30	A,V,T,M,P,L
45	G	GLY	-0.079	5	-0.586,0.179	7,4	30/30	G,E,S,T,K,R,Q
46	K	LYS	0.103	5	-0.430,0.474	6,3	30/30	D,A,K,Q,P
47	G	GLY	-1.166	9	-1.530,-1.039	9,8	30/30	G
48	G	GLY	-1.166	9	-1.530,-1.039	9,8	30/30	G
49	E	GLU	0.617	3*	-0.058,1.140	5,1	30/30	Q,D,E,T
50	V	VAL	0.025	5	-0.430,0.318	6,4	30/30	V,F,I,L
51	T	THR	-0.891	8	-1.212,-0.727	9,7	30/30	S,T
52	F	PHE	0.914	2	0.179,1.496	4,1	30/30	F,M,Y,L
53	A	ALA	-0.244	6	-0.658,0.055	7,5	30/30	T,V,A
54	V	VAL	0.274	4	-0.258,0.656	6,3	30/30	V,I,S,L,M
55	T	THR	-0.851	8	-1.154,-0.658	9,7	30/30	T,S,E,G
56	R	ARG	-1.019	8	-1.327,-0.857	9,8	30/30	K,R
57	D	ASP	-0.427	6	-0.793,-0.162	8,6	30/30	D,E,T,N
58	I	ILE	-0.664	7	-1.039,-0.430	8,6	30/30	M,I,V
59	T	THR	1.973	1	0.872,3.442	2,1	30/30	Q,T,E,D,P,K,V,A
60	N	ASN	-0.943	8	-1.212,-0.793	9,8	30/30	N,A,D,G
61	W	TRP	-1.15	9	-1.452,-0.979	9,8	30/30	W
62	N	ASN	-1.279	9	-1.530,-1.154	9,9	30/30	L,N
63	V	VAL	0.412	4*	-0.162,0.872	6,2	30/30	L,P,I,T,V
64	T	THR	1.803	1	0.872,2.041	2,1	30/30	L,N,V,I,T
65	S	SER	-1.255	9	-1.452,-1.154	9,9	30/30	G,S
66	A	ALA	0.25	4	-0.258,0.656	6,3	30/30	N,S,A,G
67	L	LEU	0.061	5	-0.430,0.474	6,3	30/30	K,N,L,D,A,E
68	G	GLY	-0.244	6*	-0.793,0.179	8,4	30/30	G,I,T
69	N	ASN	-1.12	9	-1.388,-0.979	9,8	30/30	N,G,D
70	N	ASN	-0.395	6	-0.793,-0.162	8,6	30/30	N,V,I,T

71	A	ALA	0.425	4*	-0.258,0.872	6,2	29/30	D,S,A,V,F,Q,L
72	V	VAL	-0.265	6	-0.727,0.055	7,5	30/30	V,A,E,W,D
73	V	VAL	0.26	4	-0.258,0.656	6,3	30/30	Y,N,V,I,T,A,S
74	R	ARG	1.255	1	0.318,2.041	4,1	30/30	V,A,S,G,N,Q,R
75	T	THR	1.061	2	0.318,1.496	4,1	30/30	M,S,L,E,W,H,T,F
76	V	VAL	-0.246	6	-0.658,0.055	7,5	30/30	V,T,A,S
77	T	THR	-0.027	5	-0.511,0.318	7,4	30/30	A,T,I,L
78	L	LEU	0.435	4*	-0.162,0.872	6,2	30/30	R,Q,K,L,G,D,F
79	P	PRO	1.338	1	0.474,2.041	3,1	30/30	L,P,T,V,E,A,G
80	I	ILE	1.661	1	0.656,2.041	3,1	30/30	T,I,F,V,Y,L
81	V	VAL	0.372	4*	-0.258,0.872	6,2	30/30	L,Y,V,D,S
82	P	PRO	-0.968	8	-1.327,-0.793	9,8	30/30	T,P
83	S	SER	0.694	3*	0.055,1.140	5,1	30/30	F,H,G,S,A,Y,R
84	A	ALA	-0.675	7	-1.039,-0.430	8,6	30/30	A,V,T
85	F	PHE	-1.007	8	-1.327,-0.793	9,8	30/30	H,F
87	V	VAL	0.908	2	0.179,1.496	4,1	30/30	V,I,T,A,S,Y,N
88	Q	GLN	1.189	1	0.474,1.496	3,1	30/30	Q,L,E,T,N,K,Y,S
89	P	PRO	-1.222	9	-1.530,-1.097	9,9	30/30	P
90	D	ASP	-0.974	8	-1.269,-0.793	9,8	30/30	E,D
91	F	PHE	0.166	4	-0.430,0.656	6,3	30/30	L,G,F
92	T	THR	0.734	3*	0.055,1.140	5,1	30/30	T,S,K
93	L	LEU	1.913	1	0.872,3.442	2,1	30/30	T,F,V,S,P,L
94	K	LYS	1.52	1	0.656,2.041	3,1	30/30	V,T,E,A,K,Q
96	N	ASN	-1.274	9	-1.530,-1.154	9,9	30/30	A,N
97	T	THR	0.356	4	-0.162,0.656	6,3	30/30	T,S,E,N,K
98	D	ASP	-0.943	8	-1.269,-0.727	9,7	30/30	N,A,D
99	L	LEU	-0.071	5	-0.586,0.318	7,4	30/30	V,F,L
100	L	LEU	-0.44	6	-0.857,-0.162	8,6	30/30	M,L,V,F
101	E	GLU	0.771	3	0.179,1.140	4,1	30/30	V,I,T,D,E,A
102	S	SER	-1.396	9	-1.589,-1.327	9,9	30/30	S
103	A	ALA	-1.198	9	-1.452,-1.097	9,9	30/30	V,A
104	E	GLU	0.081	5	-0.430,0.474	6,3	30/30	E,D,T,Q,K
105	L	LEU	2.203	1	1.140,3.442	1,1	30/30	L,Q,M,I,V,A
106	T	THR	0.003	5	-0.511,0.318	7,4	28/30	T,V
107	S	SER	-0.858	8	-1.154,-0.658	9,7	21/30	N,S
108	T	THR	1.179	1	0.318,1.496	4,1	23/30	S,V,K,E,D,G,T,Q
109	D	ASP	0.523	3	-0.058,0.872	5,2	30/30	N,D,G,S
110	P	PRO	-0.933	8	-1.327,-0.727	9,7	30/30	P,V
111	Q	GLN	-0.433	6	-0.793,-0.162	8,6	30/30	Q,M,T,E
112	T	THR	-0.477	7	-0.857,-0.258	8,6	30/30	T,V
113	V	VAL	0.298	4	-0.258,0.656	6,3	30/30	L,I,V
114	V	VAL	-0.211	6	-0.658,0.055	7,5	30/30	T,V,E
115	Y	TYR	-1.266	9	-1.530,-1.154	9,9	30/30	Y

116	R	ARG	-0.21	6	-0.658,0.055	7,5	30/30	R,K,E,T,V
117	I	ILE	-1.055	8	-1.327,-0.919	9,8	30/30	L,I,V
118	R	ARG	0.222	4	-0.347,0.656	6,3	30/30	N,Q,R,K
119	E	GLU	1.255	1	0.474,2.041	3,1	30/30	E,T,Q,K,P
120	D	ASP0	1.44	1	0.656,2.041	3,1	30/30	N,Q,K,A,E,D
121	A	ALA	-1.358	9	-1.589,-1.269	9,9	30/30	A
122	V	VAL	-0.011	5	-0.511,0.318	7,4	30/30	K,Q,V,T,S,A
123	W	TRP	-1.15	9	-1.452,-0.979	9,8	30/30	W
124	S	SER	0.117	5	-0.347,0.474	6,3	30/30	N,S,A,D
125	D	ASP	-1.334	9	-1.589,-1.269	9,9	30/30	D
126	G	GLY	-1.166	9	-1.530,-1.039	9,8	30/30	G
127	V	VAL	-0.473	7	-0.857,-0.258	8,6	30/30	K,T,V
128	P	PRO	-1.222	9	-1.530,-1.097	9,9	30/30	P
129	I	ILE	-0.358	6	-0.793,-0.058	8,5	30/30	I,V,F,L
130	T	THR	0.528	3	-0.058,0.872	5,2	30/30	N,T,A,S,D,G
131	G	GLY	-0.748	7	-1.097,-0.511	9,7	30/30	G,A,V
132	D	ASP	0.569	3	-0.058,0.872	5,2	30/30	S,A,E,D,P,N
133	D	ASP	-1.334	9	-1.589,-1.269	9,9	30/30	D
134	F	PHE	-1.262	9	-1.530,-1.154	9,9	30/30	F
135	I	ILE	1.061	2	0.318,1.496	4,1	30/30	V,I,D,A,Q
136	Y	TYR	-0.749	7	-1.154,-0.511	9,7	30/30	Y,L,M
137	F	PHE	0.881	2	0.179,1.496	4,1	30/30	T,F,G,L,Q,I,V,A,S,Y,N
138	W	TRP	-0.546	7	-1.039,-0.258	8,6	30/30	Y,R,W
139	K	LYS	-0.851	8	-1.154,-0.658	9,7	30/30	K,Q,R
140	T	THR	1.022	2	0.318,1.496	4,1	30/30	L,M,T,V,S,A,W
141	Q	GLN	-0.425	6	-0.793,-0.162	8,6	30/30	L,M,Q,S,E
142	N	ASN	-0.982	8	-1.269,-0.793	9,8	30/30	T,S,D,N
143	R	ARG	1.515	1	0.474,2.041	3,1	30/30	P,R,T,S,A,G
144	R	ARG	0.276	4	-0.258,0.656	6,3	30/30	A,E,T,R,K
145	D	ASP	-0.299	6	-0.727,0.055	7,5	30/30	Q,E,D,T
146	C	CYS	-1.163	9	-1.530,-0.979	9,8	30/30	C
147	P	PRO	0.742	3*	0.055,1.140	5,1	30/30	T,A,P,K
148	E	GLU	2.198	1	1.140,3.442	1,1	30/30	T,A,E,G,D,K
149	C	CYS	-1.163	9	-1.530,-0.979	9,8	30/30	C
150	L	LEU	1.013	2	0.318,1.496	4,1	30/30	P,L,K,T,E,A,S
151	I	ILE	0.566	3	-0.058,0.872	5,2	30/30	V,T,I,A,S,P
152	N	ASN	-0.99	8	-1.269,-0.793	9,8	30/30	A,G,N
153	A	ALA	0.899	2	0.179,1.496	4,1	30/30	R,H,G,D,S,A,T
154	S	SER	-0.546	7	-0.919,-0.347	8,6	30/30	P,N,T,S
155	Y	TYR	1.596	1	0.656,2.041	3,1	30/30	M,Y,A,S,D,G
156	G	GLY	-1.166	9	-1.530,-1.039	9,8	30/30	G
157	H	HIS	-0.045	5	-0.586,0.318	7,4	30/30	F,W,H,L,Y
158	D	ASP	-0.321	6	-0.727,-0.058	7,5	30/30	N,K,E,D

159	F	PHE	0.709	3*	0.055,1.140	5,1	30/30	L,Q,F,D,R,M,K,V
160	I	ILE	0.317	4	-0.258,0.656	6,3	30/30	L,A,I,V
161	E	GLU	3.11	1	2.041,3.442	1,1	30/30	D,E,T,Q,S,A,K,R,N
162	T	THR	-0.209	6	-0.658,0.055	7,5	30/30	T,D,A,S,K,N
163	L	LEU	-0.204	6	-0.658,0.055	7,5	30/30	L,I,V
164	E	GLU	-0.056	5	-0.511,0.318	7,4	30/30	V,T,I,D,E
165	Q	GLN	-0.188	6*	-0.793,0.179	8,4	30/30	G,Q,P,L
166	D	ASP6	-0.912	8	-1.212,-0.727	9,7	30/30	Q,T,D,S
167	E	GLU	-0.704	7	-1.097,-0.430	9,6	30/30	A,E,D,N
168	T	THR	0.405	4*	-0.162,0.872	6,2	30/30	G,D,T,N
169	G	GLY	-0.873	8	-1.269,-0.658	9,7	30/30	K,G
170	K	LYS	-1.026	8	-1.327,-0.857	9,8	30/30	R,K
171	V	VAL	-1.207	9	-1.452,-1.097	9,9	30/30	V,T
172	V	VAL	-1.003	8	-1.269,-0.857	9,8	30/30	V,I,A
173	T	THR	-1.034	8	-1.327,-0.857	9,8	30/30	T,V,K
174	A	ALA	-0.381	6	-0.793,-0.058	8,5	30/30	M,L,A,V
175	T	THR	-0.488	7	-0.857,-0.258	8,6	30/30	K,A,V,T
176	F	PHE	0.474	3*	-0.162,0.872	6,2	30/30	F,Y,L,M
177	S	SER	0.945	2	0.318,1.496	4,1	30/30	T,D,E,A,S,K,Q
178	E	GLU	0.569	3	-0.058,0.872	5,2	30/30	T,H,E,A,K,Q,R
179	P	PRO	-0.591	7	-1.039,-0.258	8,6	30/30	K,N,P
180	F	PHE	-0.018	5	-0.586,0.318	7,4	30/30	F,Y
181	L	LEU	-0.145	5	-0.586,0.179	7,4	30/30	L,P,T,V,S,A,G
182	G	GLY	-0.709	7	-1.097,-0.511	9,7	30/30	E,G,D
183	W	TRP	-1.15	9	-1.452,-0.979	9,8	30/30	W
184	Q	GLN	0.028	5	-0.430,0.318	6,4	30/30	Q,R,K
185	G	GLY	1.654	1	0.656,2.041	3,1	30/30	G,L,Q,V,A,S,N,R,K
186	L	LEU	0.526	3*	-0.162,0.872	6,2	30/30	F,G,L,Y,M
187	F	PHE	-0.436	6	-0.919,-0.162	8,6	30/30	W,F
189	F	PHE	0.288	4	-0.347,0.656	6,3	30/30	P,R,N,F,A,G
190	L	LEU	-0.033	5	-0.511,0.318	7,4	30/30	I,M,L
191	Y	TYR	-0.415	6	-0.857,-0.058	8,5	30/30	Y,L,F
192	P	PRO	-1.222	9	-1.530,-1.097	9,9	30/30	P
193	A	ALA	-1.017	8	-1.327,-0.857	9,8	30/30	S,A,G
194	H	HIS4	-1.337	9	-1.589,-1.269	9,9	30/30	H
195	L	LEU	-0.616	7	-0.979,-0.347	8,6	30/30	L,V,I
196	A	ALA	-1.194	9	-1.452,-1.039	9,8	30/30	G,A
197	E	GLU	0.523	3	-0.058,0.872	5,2	30/30	K,R,A,E
198	K	LYS	-0.488	7	-0.857,-0.258	8,6	30/30	E,K,Q
199	H	HIS	0.341	4	-0.162,0.656	6,3	30/30	H,Q,N,L
200	G	GLY	-1.166	9	-1.530,-1.039	9,8	30/30	G
201	D	ASP	-0.185	6	-0.727,0.179	7,4	30/30	S,G,D,T
202	I	ILE	-0.594	7	-0.979,-0.347	8,6	30/30	I,L

203	A	ALA	-0.614	7	-0.979,-0.347	8,6	30/30	K,Y,A
204	E	GLU	1.663	1	0.656,2.041	3,1	30/30	K,T,A,S,E,D
205	S	SER	-0.819	8	-1.097,-0.658	9,7	30/30	A,S
206	Y	TYR	-0.041	5	-0.586,0.318	7,4	30/30	Y,F
207	N	ASN	1.057	2	0.318,1.496	4,1	30/30	D,S,A,E,T,K,N
208	N	ASN	0.596	3*	-0.347,1.140	6,1	4/30	E,D,G,N
209	F	PHE	0.396	4*	-0.258,0.872	6,2	30/30	R,Y,W,F
210	L	LEU	-0.033	5	-0.586,0.318	7,4	30/30	F,L,Y
211	S	SER	1.101	1	0.318,1.496	4,1	30/30	V,E,S,D,G,Q
212	N	ASN	2.09	1	0.872,3.442	2,1	30/30	D,E,T,Q,S,A,K,N
213	E	GLU	-0.373	6	-0.793,-0.058	8,5	30/30	S,E,T,N
214	V	VAL	-0.367	6	-0.793,-0.058	8,5	30/30	A,F,V,M,P
215	P	PRO	-1.222	9	-1.530,-1.097	9,9	30/30	P
216	A	ALA	0.237	4	-0.258,0.656	6,3	30/30	N,E,A,D,T,V
217	W	TRP	-0.077	5	-0.658,0.318	7,4	30/30	F,W,Y
218	S	SER	-0.844	8	-1.154,-0.658	9,7	30/30	S,T
219	G	GLY	-0.016	5	-0.586,0.318	7,4	30/30	A,G
220	G	GLY	-0.339	6	-0.857,-0.058	8,5	30/30	N,A,S,G
221	P	PRO	-0.736	7	-1.154,-0.511	9,7	30/30	P,A
222	Y	TYR	0.774	3*	0.055,1.140	5,1	30/30	F,W,Y
224	V	VAL	-0.518	7	-0.919,-0.258	8,6	30/30	I,V,F
225	E	GLU	2.068	1	1.140,3.442	1,1	30/30	T,E,G,D,Q,A,S,K
226	S	SER	0.036	5	-0.430,0.318	6,4	30/30	K,N,D,A,S
227	F	PHE	0.234	4	-0.347,0.656	6,3	30/30	Y,A,W,F
228	D	ASP	0.937	2	0.318,1.496	4,1	30/30	Q,N,K,V,E,D
229	P	PRO	0.605	3*	-0.058,1.140	5,1	30/30	D,A,T,K,N,P
230	G	GLY	-0.566	7	-0.919,-0.347	8,6	30/30	K,N,D,G
231	Q	GLN	1.77	1	0.872,2.041	2,1	30/30	K,R,V,I,Q,E,F,T
232	L	LEU	-0.201	6	-0.658,0.055	7,5	30/30	Q,L,S,A
233	V	VAL	0.105	5	-0.430,0.474	6,3	30/30	A,V,I,L
234	T	THR	-1.202	9	-1.452,-1.097	9,9	30/30	V,T
235	L	LEU	0.629	3*	-0.058,1.140	5,1	30/30	A,E,T,M,L
236	V	VAL	-0.408	6	-0.793,-0.162	8,6	30/30	K,V,A,E
237	P	PRO	-0.39	6	-0.857,-0.058	8,5	30/30	Q,R,P
238	N	ASN	-1.403	9	-1.589,-1.327	9,9	30/30	N
239	P	PRO	0.001	5	-0.511,0.318	7,4	30/30	P,E,D
240	K	LYS	0.434	4*	-0.162,0.872	6,2	30/30	Q,N,R,K,E,A,T
241	W	TRP	0.92	2	0.179,1.496	4,1	30/30	Y,W,F
242	Y	TYR	-0.381	6	-0.857,-0.058	8,5	30/30	W,Y
243	G	GLY	-1.166	9	-1.530,-1.039	9,8	30/30	G
244	E	GLU	2.71	1	1.496,3.442	1,1	30/30	Q,K,S,A,E,D,T
245	K	LYS	0.129	5*	-0.586,0.656	7,3	12/30	T,E,Q,K
246	G	GLY	0.487	3*	-0.162,0.872	6,2	30/30	A,G,Q,R,K,P

247	P	PRO	-0.383	6	-0.857,-0.058	8,5	30/30	P,S,A,V
248	Y	TYR	1.334	1	0.474,2.041	3,1	30/30	T,H,Y,P,K,R,N,A,S
249	L	LEU	-1.037	8	-1.388,-0.857	9,8	30/30	V,L
250	D	ASP	0.424	4*	-0.162,0.872	6,2	30/30	E,S,A,G,D,Q,N
251	K	LYS	0.101	5	-0.430,0.474	6,3	30/30	A,S,T,Q,R,K
252	L	LEU	0.49	3*	-0.162,0.872	6,2	30/30	L,I,V
253	K	LYS	0.48	3	-0.058,0.872	5,2	30/30	K,V,T,I
254	F	PHE	-0.128	5	-0.658,0.179	7,4	30/30	M,L,Y,F,I
255	R	ARG	-1.147	9	-1.452,-0.979	9,8	30/30	S,R
256	I	ILE	-0.157	6	-0.586,0.179	7,4	30/30	M,V,I
257	I	ILE	-1.228	9	-1.452,-1.097	9,9	30/30	V,I
258	T	THR	-1.365	9	-1.589,-1.269	9,9	30/30	T
259	D	ASP	-0.719	7	-1.097,-0.511	9,7	30/30	K,D,E
260	S	SER	-0.074	5	-0.586,0.318	7,4	30/30	A,S,D,G,T,Q,P
261	T	THR	0.805	2	0.179,1.140	4,1	30/30	G,S,A,T,N,Q
262	Q	GLN	-0.967	8	-1.269,-0.793	9,8	30/30	E,Q,L
263	Q	GLN	-0.66	7	-1.039,-0.430	8,6	30/30	L,Q,T,E
264	L	LEU	-0.116	5	-0.658,0.318	7,4	30/30	V,M,L,P
265	T	THR	0.165	4	-0.347,0.474	6,3	30/30	P,L,V,I,T,S
266	A	ALA	-1.358	9	-1.589,-1.269	9,9	30/30	A
267	L	LEU	-0.82	8	-1.212,-0.586	9,7	30/30	L,M
268	E	GLU	-0.806	8	-1.097,-0.586	9,7	30/30	E,A,R,Q
269	N	ASN	-1.403	9	-1.589,-1.327	9,9	30/30	N
270	G	GLY	-0.31	6	-0.727,-0.058	7,5	30/30	D,G,R,N
271	E	GLU	-1.376	9	-1.589,-1.327	9,9	30/30	E
272	V	VAL	-1.187	9	-1.452,-1.039	9,8	30/30	I,V
273	D	ASP3	-0.677	7	-1.039,-0.430	8,6	30/30	D,N,Q
274	V	VAL	-0.084	5	-0.586,0.179	7,4	30/30	A,V,I
275	I	ILE	-0.469	7	-0.857,-0.258	8,6	30/30	V,I,L,M
276	Y	TYR	-0.507	7	-0.919,-0.258	8,6	30/30	F,A,Y,N
277	P	PRO	-1.222	9	-1.530,-1.097	9,9	30/30	P
278	Q	GLN	-1.011	8	-1.269,-0.857	9,8	30/30	D,I,Q
279	G	GLY	-0.913	8	-1.269,-0.658	9,7	30/30	P,G
280	A	ALA	-0.581	7	-0.919,-0.347	8,6	30/30	Q,N,T,A,S
281	T	THR	-0.773	7	-1.097,-0.586	9,7	30/30	P,T,V,A
282	Q	GLN	-0.703	7*	-1.327,-0.347	9,6	3/30	Q
283	D	ASP	-1.334	9	-1.589,-1.269	9,9	30/30	D
285	V	VAL	-0.585	7	-0.979,-0.347	8,6	30/30	V,I,L
286	E	GLU	0.414	4*	-0.162,0.872	6,2	30/30	S,K,R,N,D,E,T,Q
287	Q	GLN	-1.255	9	-1.452,-1.154	9,9	30/30	Q,N
288	A	ALA	0.399	4*	-0.162,0.872	6,2	30/30	A,V,T,I,L
289	A	ALA	0.889	2	0.179,1.496	4,1	30/30	Q,T,G,D,R,N,K,A
290	G	GLY	0.499	3	-0.058,0.872	5,2	30/30	A,D,G,Q,R,N

291	L	LEU	-0.572	7	-0.919,-0.347	8,6	30/30	L,M,I
292	D	ASP	0.803	2*	0.055,1.140	5,1	30/30	T,G,D,S,P,Q
293	Y	TYR	0.883	2*	-0.162,1.496	6,1	4/30	Y,Q,S
294	L	LEU	0.632	3*	-0.347,1.140	6,1	4/30	Q,M,L
295	G	GLY	0.022	5	-0.430,0.318	6,4	30/30	D,G,E,N
296	I	ILE	-0.262	6	-0.727,0.055	7,5	30/30	A,V,T,I
297	D	ASP	-0.445	6	-0.857,-0.162	8,6	30/30	H,D,S,K,N,Q
298	F	PHE	-0.384	6	-0.793,-0.058	8,5	30/30	Q,N,Y,S,W,F
299	Q	GLN	1.751	1	0.656,2.041	3,1	30/30	L,Q,F,T,H,Y,N,V,A
301	N	ASN	-0.314	6	-0.793,0.055	8,5	30/30	A,G,V,N,Q
302	P	PRO	0.748	3*	0.055,1.140	5,1	30/30	L,Q,E,P,K,M,A,S
303	S	SER	-0.241	6	-0.793,0.055	8,5	30/30	A,S,G
304	A	ALA	-0.793	8	-1.154,-0.586	9,7	30/30	L,I,A
305	N	ASN	0.302	4	-0.258,0.656	6,3	30/30	S,V,T,N,R,Q
306	W	TRP	-0.399	6	-0.919,-0.058	8,5	30/30	M,K,W
307	Y	TYR	-1.023	8	-1.327,-0.857	9,8	30/30	Y,Q,E
308	F	PHE	-0.628	7	-1.039,-0.347	8,6	30/30	M,F,A,H
310	G	GLY	-0.313	6	-0.793,0.055	8,5	30/30	L,A,D,G,V
311	L	LEU	0.274	4	-0.347,0.656	6,3	30/30	F,M,P,L
312	N	ASN	-1.403	9	-1.589,-1.327	9,9	30/30	N
313	S	SER	-0.109	5	-0.658,0.318	7,4	30/30	T,H,S,L,M
314	K	LYS	2.253	1	1.140,3.442	1,1	30/30	V,A,S,N,R,K,D,Q
315	A	ALA	0.036	5	-0.430,0.318	6,4	30/30	S,A,G,T,N
316	G	GLY	0.985	2	0.179,1.496	4,1	30/30	T,G,S,A,E,P,K
317	P	PRO	1.421	1	0.474,2.041	3,1	30/30	Y,P,A,F,V
319	S	SER	2.493	1	1.140,3.442	1,1	30/30	A,S,G,R,N,Q,K
320	D	ASP	0.015	5	-0.511,0.318	7,4	30/30	N,Q,D,E,S
321	I	ILE	1.177	1	0.474,1.496	3,1	30/30	A,V,I,K,P,E,T,L
322	A	ALA	-0.367	6	-0.793,-0.058	8,5	30/30	P,K,T,A
323	L	LEU	-0.773	7	-1.154,-0.511	9,7	30/30	L,I,V
324	R	ARG	-1.332	9	-1.589,-1.212	9,9	30/30	R
325	K	LYS	0.32	4	-0.258,0.656	6,3	30/30	Q,K,A,D,T
326	A	ALA	-1.358	9	-1.589,-1.269	9,9	30/30	A
327	V	VAL	0.814	2	0.179,1.140	4,1	30/30	L,M,I,V
328	L	LEU	0.583	3*	-0.058,1.140	5,1	30/30	Y,L,M,F,A
329	T	THR	-1.211	9	-1.452,-1.097	9,9	30/30	T,I
330	A	ALA	-1.2	9	-1.452,-1.097	9,9	30/30	S,A
331	I	ILE	-0.044	5	-0.511,0.318	7,4	30/30	V,I,T,M
332	D	ASP	-0.454	6	-0.857,-0.162	8,6	30/30	S,G,D,N
333	A	ALA	0.771	3*	0.055,1.140	5,1	30/30	A,V,T,K,R,Q
334	G	GLY	0.315	4	-0.258,0.656	6,3	30/30	S,E,G,D,Q,K
335	D	ASP	1.226	1	0.474,1.496	3,1	30/30	A,S,E,D,G,Q,N
336	L	LEU	0.171	4	-0.347,0.474	6,3	30/30	L,M,I,T,V

337	K	LYS	-1.209	9	-1.452,-1.097	9,9	30/30	K,I
338	A	ALA	0.52	3	-0.058,0.872	5,2	30/30	T,A,S,D,G,N
339	K	LYS	-0.839	8	-1.154,-0.658	9,7	30/30	G,R,K
340	T	THR	-1.365	9	-1.589,-1.269	9,9	30/30	T
341	A	ALA	-1.013	8	-1.327,-0.857	9,8	30/30	I,V,A
342	D	ASP	-0.897	8	-1.269,-0.658	9,7	30/30	G,D
343	P	PRO	-0.951	8	-1.212,-0.793	9,8	30/30	Q,P,S,T
344	Y	TYR	-0.794	8	-1.154,-0.586	9,7	30/30	Y,F
345	L	LEU	0.049	5	-0.430,0.318	6,4	30/30	L,N,T,V,A,S,D
346	R	ARG	1.051	2	0.318,1.496	4,1	30/30	N,R,K,P,S,E,D,T
347	N	ASN	0.458	4*	-0.162,0.872	6,2	30/30	D,G,S,E,K,N
348	W	TRP	0.12	5	-0.430,0.474	6,3	30/30	V,I,T,W,A,L
349	P	PRO	2.137	1	1.140,3.442	1,1	30/30	K,N,P,S,A,Q,G,E,T
350	H	HIS	-0.696	7	-1.097,-0.430	9,6	30/30	H,A,P
352	G	GLY	0.24	4	-0.258,0.656	6,3	30/30	D,H,G,T,R,Q,N
353	S	SER	-0.84	8	-1.154,-0.658	9,7	30/30	N,S
354	V	VAL	0.336	4	-0.258,0.656	6,3	30/30	L,H,G,R,N,K,V,A
356	F	PHE	-0.777	7	-1.154,-0.511	9,7	30/30	I,F,Y
357	L	LEU	-0.129	5	-0.586,0.179	7,4	30/30	V,M,L
358	P	PRO	-0.958	8	-1.327,-0.727	9,7	30/30	P,Q
359	N	ASN	0.395	4*	-0.162,0.872	6,2	30/30	G,D,S,Q,N
360	Q	GLN	-0.976	8	-1.269,-0.793	9,8	30/30	E,M,Q
361	A	ALA	2.986	1	1.496,3.442	1,1	30/30	Q,T,D,E,P,K,N,V,A
362	G	GLY	-0.626	7	-1.097,-0.347	9,6	30/30	A,E,G
363	Y	TYR	-1.041	8	-1.388,-0.857	9,8	30/30	Y,F
364	A	ALA	1.395	1	0.656,2.041	3,1	30/30	K,Q,V,T,E,A
365	D	ASP5	-1.16	9	-1.452,-1.039	9,8	30/30	N,D
366	R	ARG	-0.165	6	-0.658,0.179	7,4	30/30	L,N,R,T,V,H
367	R	ARG	1.618	1	0.656,2.041	3,1	30/30	T,E,L,Q,I,V,S,R
368	G	GLY	0.832	2	0.179,1.140	4,1	30/30	Q,G,D,S,A,V,T
369	A	ALA	0.771	3*	0.055,1.140	5,1	30/30	T,E,D,G,Q,S,A,K
370	R	ARG	0.525	3*	-0.162,0.872	6,2	30/30	L,Y,R,T,F,S,H
371	G	GLY	0.222	4	-0.430,0.656	6,3	29/30	S,D,G,N,K
372	Y	TYR	0.73	3*	0.055,1.140	5,1	29/30	Q,Y,L,H,W,F,I
373	G	GLY	-1.166	9	-1.530,-1.039	9,8	30/30	G
374	T	THR	0.875	2	0.179,1.140	4,1	30/30	N,L,A,S,T
375	G	GLY	-1.166	9	-1.530,-1.039	9,8	30/30	G
376	D	ASP	-0.387	6	-0.793,-0.058	8,5	30/30	N,D
377	V	VAL	1.282	1	0.474,1.496	3,1	30/30	L,V,I,T
378	E	GLU	-0.149	5	-0.586,0.179	7,4	30/30	D,A,E,N
379	K	LYS	1.31	1	0.474,2.041	3,1	30/30	K,R,T,E,A
380	A	ALA	-1.358	9	-1.589,-1.269	9,9	30/30	A
381	K	LYS1	-0.681	7	-1.039,-0.430	8,6	30/30	R,K,E

382	G	GLY	0.435	4*	-0.162,0.872	6,2	30/30	Q,K,A,E,S,G
383	I	ILE	-0.908	8	-1.212,-0.727	9,7	30/30	M,L,V,I
384	L	LEU	-1.264	9	-1.530,-1.154	9,9	30/30	L
385	S	SER	-0.657	7	-1.039,-0.430	8,6	30/30	Q,I,T,A,S
386	E	GLU	0.362	4	-0.162,0.656	6,3	30/30	Q,T,D,S,E
387	A	ALA	-1.358	9	-1.589,-1.269	9,9	30/30	A
388	G	GLY	-1.166	9	-1.530,-1.039	9,8	30/30	G
389	Y	TYR	-1.266	9	-1.530,-1.154	9,9	30/30	Y
390	K	LYS	-0.069	5	-0.511,0.318	7,4	30/30	R,Q,K,E,T
391	L	LEU	2.144	1	1.140,3.442	1,1	30/30	G,I,V,Q,L
392	S	SER	2.169	1	1.140,3.442	1,1	30/30	A,S,V,K,N,D,E,T,Q
393	G	GLY	-0.295	6	-0.793,0.055	8,5	30/30	N,D,G
394	G	GLY	3.138	1	2.041,3.442	1,1	30/30	T,D,G,S,E,N,Q
395	S	SER	1.258	1	0.474,1.496	3,1	30/30	T,D,S,A,K,R,Q
396	L	LEU	-1.264	9	-1.530,-1.154	9,9	30/30	L
397	L	LEU	2.488	1	1.140,3.442	1,1	29/30	L,Q,T,F,M,K,I,V,S
398	D	ASP	1.204	1	0.474,1.496	3,1	30/30	N,R,T,D,A
399	P	PRO	-0.383	6	-0.857,-0.058	8,5	30/30	P,K
400	S	SER	1.789	1	0.872,2.041	2,1	30/30	K,Q,N,I,D,E,S
401	G	GLY	-1.166	9	-1.530,-1.039	9,8	30/30	G
402	K	LYS	2.111	1	1.140,3.442	1,1	30/30	T,E,A,L,K,Q,N
403	P	PRO	2.241	1	1.140,3.442	1,1	30/30	V,T,D,A,P,N
404	V	VAL	-0.647	7	-1.039,-0.430	8,6	30/30	V,I
405	S	SER	-0.413	6	-0.857,-0.058	8,5	29/30	S,A,P
406	T	THR	2.923	1	1.496,3.442	1,1	30/30	Q,E,D,T,N,P,A,S
407	L	LEU	0.29	4	-0.258,0.656	6,3	30/30	M,L,I
408	R	ARG	-0.71	7	-1.097,-0.511	9,7	30/30	R,D,S,T
409	L	LEU	0.215	4	-0.347,0.656	6,3	30/30	A,I,C,M,L
410	S	SER	-0.021	5	-0.511,0.318	7,4	30/30	T,V,A,S,G,R
411	F	PHE	-0.336	6	-0.793,-0.058	8,5	30/30	F,H,Y
412	P	PRO	-0.814	8	-1.154,-0.586	9,7	30/30	P,I,T,S
413	P	PRO	0.376	4*	-0.162,0.872	6,2	30/30	Q,P,A,T,I,V
414	G	GLY	-0.872	8	-1.269,-0.658	9,7	30/30	N,G
415	Y	TYR	-1.258	9	-1.452,-1.154	9,9	30/30	N,Y
416	P	PRO	0.086	5	-0.430,0.474	6,3	30/30	P,Q,T,S,E,A
417	A	ALA	-0.271	6	-0.727,0.055	7,5	30/30	I,V,A,L
418	A	ALA	-0.619	7	-1.039,-0.347	8,6	30/30	L,R,I,A
419	N	ASN	-0.455	6	-0.857,-0.162	8,6	30/30	K,N,Q,E,S,A
420	D	ASP0	0.662	3*	0.055,1.140	5,1	30/30	D,S,V,T,Q,N
422	A	ALA	0.96	2	0.179,1.496	4,1	30/30	C,S,A,M
423	R	ARG	-0.697	7	-1.039,-0.511	8,7	30/30	K,Q,R,E
424	L	LEU	-0.784	8	-1.154,-0.511	9,7	30/30	I,V,L
425	I	ILE	-0.802	8	-1.154,-0.586	9,7	30/30	V,F,I

426	T	THR	-0.175	6	-0.658,0.179	7,4	30/30	A,G,T,Q
427	G	GLY	1.294	1	0.474,2.041	3,1	30/30	Q,D,G,T,K,N,A,S
428	Y	TYR	1.208	1	0.318,1.496	4,1	30/30	D,A,S,T,M,Q,Y
429	I	ILE	0.439	4*	-0.162,0.872	6,2	30/30	A,V,I,M,L
430	A	ALA	0.313	4	-0.258,0.656	6,3	30/30	E,S,A,K,Q,R
431	P	PRO	0.56	3	-0.058,0.872	5,2	30/30	P,K,Q,E
432	L	LEU	-1.264	9	-1.530,-1.154	9,9	30/30	L
433	G	GLY	-1.166	9	-1.530,-1.039	9,8	30/30	G
434	L	LEU	0.351	4	-0.162,0.656	6,3	30/30	I,V,M,L
435	K	LYS	0.967	2	0.318,1.496	4,1	30/30	K,N,T,D,G,E,S
436	T	THR	-0.237	6	-0.658,0.055	7,5	30/30	L,V,I,T
437	D	ASP	1.191	1	0.474,1.496	3,1	30/30	T,A,E,D,N,Q,K
438	L	LEU	0.24	4	-0.347,0.656	6,3	30/30	Y,L,I,V,F
439	L	LEU	3.256	1	2.041,3.442	1,1	30/30	L,Q,T,E,D,N,V,S,A
440	T	THR	-0.181	6	-0.658,0.179	7,4	30/30	S,T,Q,P
441	G	GLY	-0.459	6*	-1.212,-0.058	9,5	3/30	G
442	P	PRO	-1.181	9	-1.452,-1.039	9,8	30/30	P,T
443	N	ASN	-1.173	9	-1.452,-1.039	9,8	30/30	D,N
444	A	ALA	-0.614	7	-0.979,-0.347	8,6	30/30	D,S,A
445	T	THR	-0.777	7	-1.154,-0.511	9,7	30/30	T,I,L
446	A	ALA	-0.921	8	-1.269,-0.727	9,7	30/30	A,G
447	D	ASP	1.392	1	0.474,2.041	3,1	30/30	A,D,G,T,Q,K
448	Y	TYR	-1.177	9	-1.452,-1.039	9,8	30/30	Y,T
449	L	LEU	0.904	2	0.179,1.496	4,1	30/30	V,T,L,M
450	L	LEU	1.18	1	0.474,1.496	3,1	30/30	N,V,I,S,L,T,D,E
451	S	SER	1.128	1	0.474,1.496	3,1	30/30	G,E,T,K,N,R,A,S
452	G	GLY	-0.289	6	-0.857,0.055	8,5	30/30	K,G,I
453	N	ASN	-0.457	6	-0.857,-0.162	8,6	30/30	N,E,D
454	F	PHE	-0.471	7	-0.919,-0.162	8,6	30/30	F,H,Y
455	D	ASP	-1.152	9	-1.452,-0.979	9,8	30/30	D,S
456	L	LEU	0.689	3*	0.055,1.140	5,1	30/30	V,I,A,L,M
457	H	HIS	0.49	3*	-0.162,0.872	6,2	30/30	M,Y,H,F,V,I
458	L	LEU	-0.103	5	-0.586,0.179	7,4	30/30	I,V,A,L,M
459	N	ASN	-0.014	5	-0.511,0.318	7,4	30/30	F,V,S,Y,M,N
460	Y	TYR	-0.605	7	-0.979,-0.347	8,6	30/30	A,G,Y
461	F	PHE	-0.579	7	-1.039,-0.258	8,6	30/30	Y,F,W
462	S	SER	-0.645	7	-0.979,-0.430	8,6	30/30	V,T,I,S
463	Q	GLN	1.496	1	0.656,2.041	3,1	30/30	A,S,G,Q,M,L
464	Q	GLN	-0.46	6	-0.857,-0.162	8,6	30/30	N,Q,T,S
465	V	VAL	-0.677	7	-1.097,-0.430	9,6	30/30	V,T,P
466	F	PHE	0.199	4	-0.347,0.656	6,3	30/30	A,F,Y,L
467	P	PRO	0.744	3*	-0.058,1.140	5,1	30/30	I,V,A,P,R
468	A	ALA	0.338	4	-0.258,0.656	6,3	30/30	A,I,F,Y

469	V	VAL	2.105	1	0.872,3.442	2,1	30/30	V,S,A,G
470	K	LYS	0.861	2*	0.055,1.496	5,1	30/30	G,S,K,Q,N
471	A	ALA	-0.804	8	-1.154,-0.586	9,7	30/30	G,A,Y
472	G	GLY	1.764	1	0.872,2.041	2,1	30/30	I,V,A,S,R,K,F,E,G,H,L,Q
473	Q	GLN	-1.233	9	-1.452,-1.097	9,9	30/30	Q,D
474	I	ILE	1.869	1	0.872,2.041	2,1	30/30	T,I,V,A,L,R,N
475	F	PHE	0.111	5*	-0.511,0.474	7,3	30/30	F,W,Y
476	L	LEU	1.053	2	0.318,1.496	4,1	30/30	T,H,G,L,K
477	R	ARG	-0.353	6	-0.727,-0.058	7,5	30/30	R,T,I,S
478	D	ASP	2.745	1	1.496,3.442	1,1	30/30	T,G,D,S,E,K,N
479	T	THR	0.035	5	-0.430,0.318	6,4	30/30	S,A,G,T,Q
480	R	ARG	0.917	2	0.179,1.496	4,1	30/30	R,G,D,A,S,E
481	Q	GLN	0.194	4	-0.347,0.474	6,3	30/30	S,A,D,L,N,Q
482	N	ASN	-1.277	9	-1.530,-1.154	9,9	30/30	D,N
483	Y	TYR	0.713	3*	0.055,1.140	5,1	29/30	Y,F
484	F	PHE	0.28	4	-0.347,0.656	6,3	30/30	M,P,G,F,V
485	G	GLY	0.763	3*	0.055,1.140	5,1	30/30	K,Q,N,H,G,E
486	F	PHE	-0.546	7	-0.979,-0.258	8,6	30/30	Y,N,F,W
487	N	ASN	1.65	1	0.656,2.041	3,1	30/30	V,T,S,E,K,N,Q
488	D	ASP	-0.394	6	-0.793,-0.162	8,6	30/30	S,D,N
489	P	PRO	1.203	1	0.318,1.496	4,1	30/30	P,K,T,A,S,E
490	K	LYS	2.304	1	1.140,3.442	1,1	30/30	T,E,D,Q,V,A,R,K
491	I	ILE	-0.33	6	-0.793,-0.058	8,5	30/30	S,V,I,T,M
492	E	GLU	-0.973	8	-1.269,-0.793	9,8	30/30	D,E
493	E	GLU	3.323	1	2.041,3.442	1,1	30/30	K,R,A,S,Q,T,D,G,E
494	I	ILE	-0.492	7	-0.919,-0.162	8,6	30/30	I,K,M,L
495	I	ILE	-0.03	5	-0.511,0.318	7,4	30/30	L,M,V,I
496	G	GLY	1.323	1	0.474,2.041	3,1	30/30	T,D,G,S,A,N,R
497	K	LYS	-0.03	5	-0.511,0.318	7,4	30/30	K,Q,D,A,E
498	A	ALA	-1.358	9	-1.589,-1.269	9,9	30/30	A
499	A	ALA	0.289	4	-0.258,0.656	6,3	30/30	N,L,S,A,I,V
500	A	ALA	0.984	2	0.318,1.496	4,1	30/30	G,E,T,Q,S,A,N,R
501	A	ALA	-0.101	5	-0.586,0.179	7,4	30/30	Q,N,T,E,S,A
502	S	SER	-0.435	6	-0.857,-0.162	8,6	30/30	V,T,A,E,S,P
503	S	SER	-0.712	7	-1.097,-0.511	9,7	30/30	S,D
504	I	ILE	2.279	1	1.140,3.442	1,1	30/30	V,I,A,P,K,R,T,D,E,Q
505	E	GLU	0.662	3*	0.055,1.140	5,1	30/30	Q,N,E,S,A,G,T
506	E	GLU	2.062	1	0.872,3.442	2,1	30/30	K,V,A,S,L,T,E,D
507	S	SER	-0.642	7	-0.979,-0.430	8,6	30/30	R,V,S,A,G
508	A	ALA	2.418	1	1.140,3.442	1,1	30/30	T,G,H,L,I,A,S,R,N,K
509	A	ALA	0.745	3*	0.055,1.140	5,1	30/30	D,S,E,A,Q
510	I	ILE	0.843	2	0.179,1.140	4,1	30/30	H,F,I,T,K,Q,L
511	L	LEU	-0.565	7	-0.979,-0.258	8,6	30/30	I,V,L

512	S	SER	-0.685	7	-0.979,-0.511	8,7	30/30	K,N,D,S,A,I
513	E	GLU	0.449	4*	-0.162,0.872	6,2	30/30	R,Q,K,A,E,D
514	A	ALA	-1.029	8	-1.327,-0.857	9,8	30/30	V,S,A
515	D	ASP	-0.648	7	-0.979,-0.430	8,6	30/30	D,N
516	E	GLU	2.012	1	0.872,3.442	2,1	30/30	R,K,A,V,Q,E,D,H
517	L	LEU	1.385	1	0.656,2.041	3,1	30/30	V,I,L,M,Q,R
518	A	ALA	0.016	5	-0.430,0.318	6,4	30/30	M,L,A,F,V,I
520	D	ASP	1.259	1	0.474,1.496	3,1	30/30	Q,N,K,E,G,H,D
521	Y	TYR	-0.979	8	-1.269,-0.793	9,8	30/30	D,Y,N
522	A	ALA	-1.177	9	-1.452,-1.039	9,8	30/30	L,A
523	A	ALA	-0.231	6	-0.727,0.055	7,5	30/30	V,I,A,L,Y
524	L	LEU	-0.789	8	-1.154,-0.586	9,7	30/30	I,V,L
525	F	PHE	-0.779	7	-1.154,-0.511	9,7	30/30	L,M,F
526	P	PRO	-1.222	9	-1.530,-1.097	9,9	30/30	P
527	I	ILE	-0.188	6	-0.658,0.179	7,4	30/30	M,L,I
528	Y	TYR	-0.808	8	-1.154,-0.586	9,7	30/30	F,Y
529	Q	GLN	-1.396	9	-1.589,-1.327	9,9	30/30	Q
530	L	LEU	-0.73	7	-1.097,-0.511	9,7	30/30	V,K,R,L
531	P	PRO	-0.421	6	-0.919,-0.058	8,5	30/30	S,E,P
532	T	THR	-1	8	-1.269,-0.857	9,8	30/30	T,D,S
533	A	ALA	-0.158	6	-0.658,0.179	7,4	30/30	L,A,I,F
534	L	LEU	0.555	3	-0.058,0.872	5,2	30/30	L,A,I
535	I	ILE	-0.96	8	-1.269,-0.793	9,8	30/30	I,A,S
536	Y	TYR	0.921	2	0.179,1.496	4,1	30/30	Q,Y,L,A,S,T,V
537	K	LYS	0.657	3*	0.055,1.140	5,1	30/30	H,Q,S,P,Y,N,R,K
538	E	GLU	0.427	4*	-0.162,0.872	6,2	30/30	N,K,E,A,S,D
539	A	ALA	0.355	4	-0.162,0.656	6,3	30/30	T,S,A,D,N,Q,K
540	I	ILE	0.721	3*	0.055,1.140	5,1	30/30	L,Y,I,V
541	L	LEU	0.552	3	-0.058,0.872	5,2	30/30	A,G,T,V,L
542	N	ASN	-1.403	9	-1.589,-1.327	9,9	30/30	N
543	L	LEU	0.327	4	-0.258,0.656	6,3	30/30	V,I,L
544	R	ARG	-1.332	9	-1.589,-1.212	9,9	30/30	R
545	D	ASP	-0.321	6	-0.727,-0.058	7,5	30/30	D,G,A,P,N
546	N	ASN	-1.403	9	-1.589,-1.327	9,9	30/30	N
547	P	PRO	-0.495	7	-0.919,-0.258	8,6	30/30	P,A,S,G
548	N	ASN	-0.4	6	-0.793,-0.162	8,6	30/30	N,S,T
549	Q	GLN	-0.129	5	-0.658,0.179	7,4	30/30	Y,L,Q,N,S
550	L	LEU	0.099	5	-0.430,0.474	6,3	30/30	L,N,V,S,A,D,H
551	G	GLY	0.209	4	-0.430,0.656	6,3	30/30	T,A,S,G
552	P	PRO	-0.647	7	-1.097,-0.347	9,6	30/30	P,T,I
553	A	ALA	0.86	2	0.179,1.496	4,1	30/30	L,P,M,T,V,A
554	Y	TYR	-1.042	8	-1.388,-0.857	9,8	30/30	Y,T
555	N	ASN	-1.403	9	-1.589,-1.327	9,9	30/30	N

556	T	THR	1.227	1	0.474,1.496	3,1	30/30	T,I,V,A,L,M
557	A	ALA	2.607	1	1.496,3.442	1,1	30/30	E,G,H,T,Q,S,A,K
558	E	GLU	0.111	5	-0.347,0.474	6,3	30/30	D,A,E,Q
559	W	TRP	-1.15	9	-1.452,-0.979	9,8	30/30	W
560	G	GLY	-0.585	7	-1.039,-0.258	8,6	30/30	G,A
561	L	LEU	0.899	2	0.179,1.496	4,1	30/30	F,V,I,L
562	A	ALA	0.97	2	0.179,1.496	4,1	16/30	K,R,L,S,A,T
563	E	GLU	0.628	3*	-0.162,1.140	6,1	14/30	S,E,D,Q,K
564	L	LEU	-0.253	6*	-1.097,0.318	9,4	1/30	L
565	E	GLU	-0.253	6*	-1.097,0.318	9,4	1/30	E
566	H	HIS	-0.253	6*	-1.154,0.179	9,4	1/30	H
567	H	HIS	-0.253	6*	-1.154,0.179	9,4	1/30	H
568	H	HIS	-0.253	6*	-1.154,0.179	9,4	1/30	H

POS: The position of The AA in The SEQRES derived sequence
SEQ: The SEQRES derived sequence in one letter code
RESID: The ATOM derived sequence in three letter code
SCORE: The normalized conservation scores
COLOR: The color scale representing the conservation scores (9 - conserved, 1 - variable)
CONFIDENCE INTERVAL: A confidence interval assigned to each of the inferred evolutionary conservation scores
CS: Confidence interval colors; the color scale representing the lower and upper bounds of the confidence interval
MSA: The number of aligned sequences having an amino acid (non-gapped) from the overall number of sequences at each position
RESIDUE VARIETY: The residues variety at each position of the multiple sequence alignment
